# Acarbose impairs gut *Bacteroides* growth by targeting intracellular glucosidases

**DOI:** 10.1128/mbio.01506-24

**Published:** 2024-11-20

**Authors:** Haley A. Brown, Adeline L. Morris, Nicholas A. Pudlo, Ashley E. Hopkins, Eric C. Martens, Jonathan L. Golob, Nicole M. Koropatkin

**Affiliations:** 1 Department of Microbiology and Immunology, University of Michigan Medical School, Ann Arbor, Michigan, USA; 2 Department of Internal Medicine, Division of Infectious Diseases, University of Michigan Medical School, Ann Arbor, Michigan, USA; Instituto Gulbenkian de Ciência, Oeiras, Portugal

**Keywords:** acarbose, alpha-amylase, glucosidase, glycoside hydrolase family 13, glycoside hydrolase family 97, *Bacteroidota*

## Abstract

Acarbose is a type 2 diabetes medicine that prevents dietary starch breakdown into glucose by inhibiting host amylase and glucosidase enzymes. Numerous gut species in the *Bacteroides* genus enzymatically break down starch and change in relative abundance within the gut microbiome in acarbose-treated individuals. To mechanistically explain this observation, we used two model starch-degrading *Bacteroides*, *Bacteroides ovatus* (Bo), and *Bacteroides thetaiotaomicron* (Bt). Bt growth on starch polysaccharides is severely impaired by acarbose, whereas Bo growth is much less affected by the drug. The *Bacteroides* use a starch utilization system (Sus) to grow on starch. We hypothesized that Bo and Bt Sus enzymes are differentially inhibited by acarbose. Instead, we discovered that although acarbose primarily targets the Sus periplasmic GH97 enzymes in both organisms, the drug affects starch processing at multiple other points. Acarbose competes for transport through the TonB-dependent SusC proteins and binds to the Sus transcriptional regulators. Furthermore, Bo expresses a non-Sus GH97 (BoGH97D) when grown in starch with acarbose. The Bt homolog, BtGH97H, is not expressed in the same conditions, nor can overexpression of BoGH97D complement the Bt growth inhibition in the presence of acarbose. This work informs us about unexpected complexities of Sus function and regulation in *Bacteroides*, including variation between related species. Furthermore, this indicates that the gut microbiome may be a source of variable response to acarbose treatment for diabetes.

**IMPORTANCE:**

Acarbose is a type 2 diabetes medication that works primarily by stopping starch breakdown into glucose in the small intestine. This is accomplished by the inhibition of host enzymes, leading to better blood sugar control via reduced ability to derive glucose from dietary starches. The drug and undigested starch travel to the large intestine where acarbose interferes with the ability of some bacteria to grow on starch. However, little is known about how gut bacteria interact with acarbose, including microbes that can use starch as a carbon source. Here, we show that two gut species, *Bacteroides ovatus* (Bo) and *Bacteroides thetaiotaomicron* (Bt), respond differently to acarbose: Bt growth is inhibited by acarbose, while Bo growth is less affected. We reveal a complex set of mechanisms involving differences in starch import and sensing behind the different Bo and Bt responses. This indicates the gut microbiome may be a source of variable response to acarbose treatment for diabetes via complex mechanisms in common gut microbes.

## INTRODUCTION

**Fig 1:**
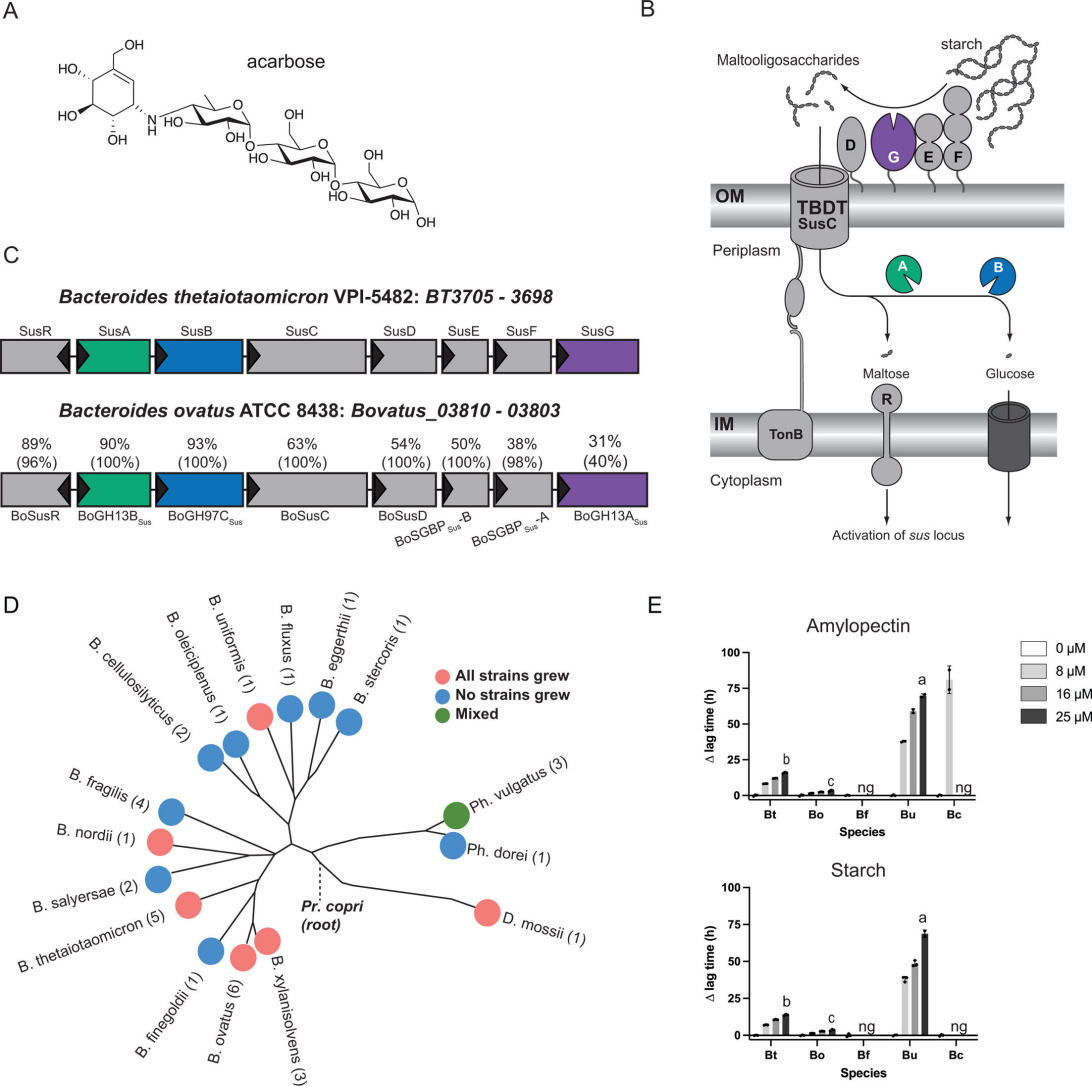
*Bacteroides* growth is inhibited by acarbose. (**A**) Chemical structure of acarbose. (**B**) Summary of the Bt starch utilization system with an emphasis on the likely enzymatic targets of acarbose: the outer member GH13 SusG, periplasmic GH97 SusB, and periplasmic GH13 SusA. (**C**) Amino acid level comparison of Bt and Bo Sus. Numbers above the Bo proteins correspond to percent identity and sequence coverage. For example, BoSusC is 63% identical to SusC over 100% of the sequence. Panels B and C were modified from references 5 and 6 with permission. (**D**) Various *Bacteroides* strains and species were grown in amylopectin or potato starch with or without 25 µM acarbose. Growth was defined as reaching an OD_600_ of 0.3 over 144 h of measured growth. The 16s rRNA-based phylogenetic tree was modified from reference 7 with permission. (**E**) Type strains of Bt, Bo, *Bacteroides fragilis* (Bf)*, Bacteroides uniformis* (Bu)*,* and *Bacteroides cellulosilyticus* (Bc) were grown in the indicated concentrations of acarbose in amylopectin and potato starch. ∆ lag time is defined as the difference in time it takes the treated and untreated conditions to reach an OD_600_ of 0.3. Statistical comparisons were performed for the 25 µM treatment only using Bo as the comparison point with a one-way ANOVA and a cutoff of *P* ≤ 0.05. Conditions with the same letter were not statistically different from one another. All growths were performed in triplicate; although in some conditions, only two replicates grew in the 144 h timeframe. The mean and standard deviation are shown in the ∆ lag graphs. ng = no growth. All species grew in the 0 µM acarbose treatment (Fig. S1 and S2).

Recent work suggests that the beneficial or toxic effects of some xenobiotics are mediated via their interactions with the gut microbiota (1). For example, the severe negative side effects of the colon cancer chemotherapeutic, CPT-11, are caused by enzymatic reactivation of the drug by symbiotic intestinal bacteria (2). Conversely, metformin, which is used to treat type 2 diabetes (T2D), elicits some of its positive effects due to a direct influence on the composition of bacteria in the gut (3). The natural product acarbose (Fig. 1A) is also FDA approved to treat T2D. Though the FDA has long known that acarbose “is metabolized exclusively within the gastrointestinal tract, principally by intestinal bacteria,” there is a paucity of data providing molecular insight into these interactions (4).

**Fig 2:**
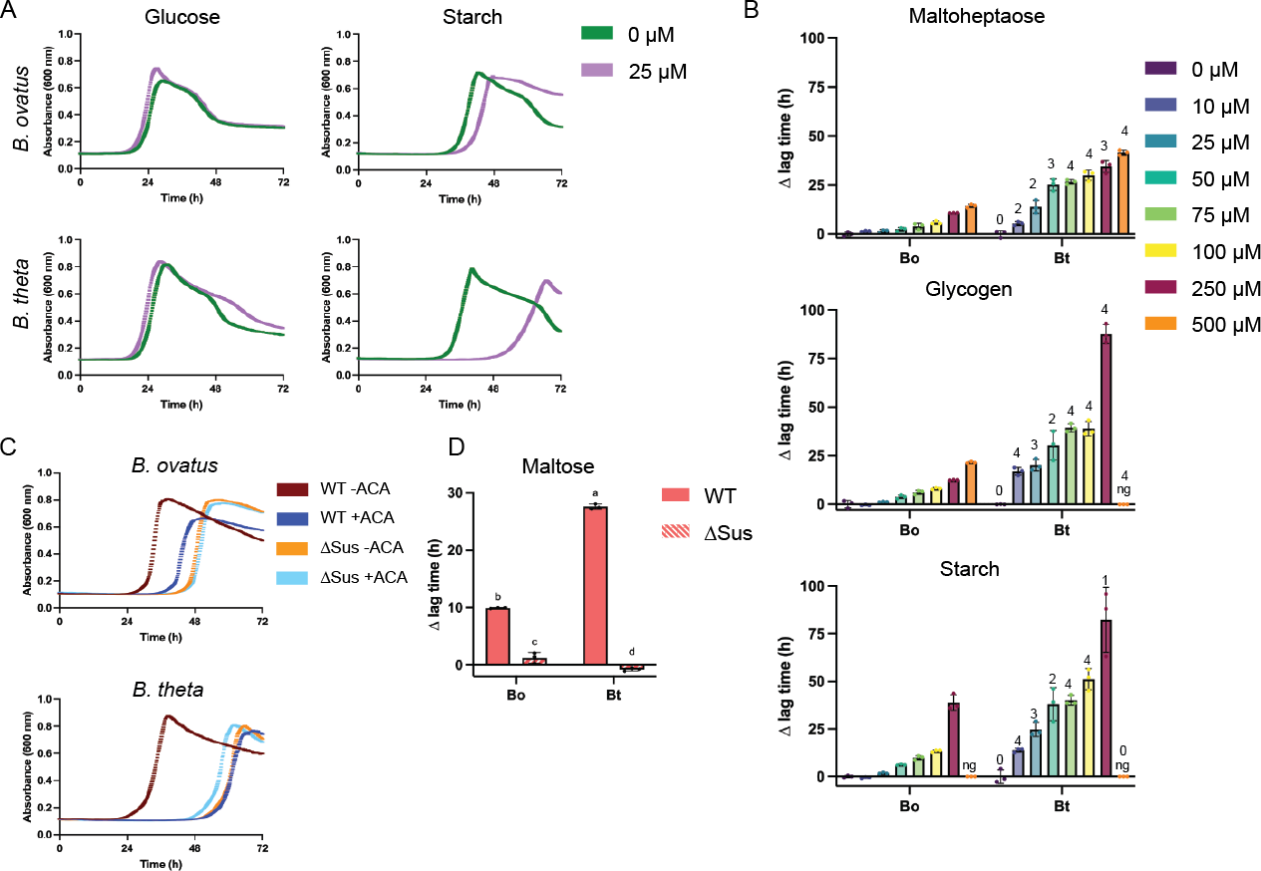
Bt is more susceptible to acarbose-induced growth inhibition than Bo. (**A**) Bo and Bt were pre-grown in minimal media (MM) with glucose and back diluted into MM + 2.5 mg/mL of the indicated carbon sources with or without 25 µM acarbose. (**B**) Bo and Bt were pre-grown as described in panel A and back diluted into MM + 2.5 mg/mL of the indicated carbon sources with and without 10–500 µM acarbose. The difference in time to OD_600_ of 0.3 between the treated and untreated conditions (∆ lag time) is graphed. Bo and Bt with the same treatment were compared using an unpaired, two-tailed Student’s *t* test. 1: *P* ≤ 0.05; 2: *P* ≤ 0.01; 3: *P* ≤ 0.001. 4: *P* ≤ 0.0001. ng = no growth. (**C**) WT and ∆Sus strains of Bo and Bt were pre-grown as described in panel A and back diluted into MM +2.5 mg/mL maltose with or without 50 µM acarbose. (**D**) ∆ lag time as described in panel B is shown from curves in panel C. Statistical analyses were performed using a two-way ANOVA with a cutoff of *P* ≤ 0.05. Conditions with the same letter were not statistically different from one another. All growths were performed in triplicate. The mean and standard deviation are shown in the ∆ lag graphs, while growth curves show the average of the three curves.

The direct function of acarbose is to prevent postprandial blood glucose spikes by inhibiting host amylases and glucosidases (collectively termed glucoamylases) used to digest starch in the upper gastrointestinal tract (8). Acarbose functions as a transition state mimic for α1,4 glycosidic bond hydrolysis to inhibit these enzymes (9). During acarbose treatment, more starch transits to the large intestine where it is bacterially fermented to short-chain fatty acids (SCFAs) (10–12). Acarbose also changes the microbially derived bile acid pool which may contribute to its anti-hypoglycemic effects by influencing host metabolic signaling (13, 14).

In addition to being an effective treatment for T2D, acarbose shows promise in ameliorating symptoms in immune and cardiovascular diseases and consistently enhances murine lifespan, likely due to altered SCFA output and the interplay between glucose metabolism and aging (15–20). Because acarbose efficacy may depend on an individual’s baseline microbiota and genetic predisposition to disease (13), molecular insight into the drug’s influence on bacterial growth and fitness is critical to repurposing this drug and improving outcomes in diseases besides T2D.

Since acarbose is minimally absorbed by host tissue and transits the gut, one explanation for its gut microbial effects is that it changes bacterial fitness by inhibiting bacterial glucoamylases (21, 22). Indeed, preliminary work shows that gut bacterial species are differentially inhibited by acarbose *in vitro* when grown in starch (23–25). In particular, members of the prevalent Bacteroidota phylum may be less fit since their relative abundance decreases in acarbose-treated individuals (13). Species in this phylum normally deploy a well-characterized Starch utilization system (Sus) to recognize, degrade, and import starch (Fig. 1B) (5). Starch is comprised of amylose and amylopectin. Amylose is made of almost exclusively α1,4-linked glucose, while amylopectin contains α1,6 branch points (26). In the model organism *Bacteroides thetaiotaomicron* (Bt), Sus is comprised of the outer membrane lipoproteins SusDEF that bind starch and a lipoprotein α-amylase, SusG (27–36). Maltooligosaccharides are transported to the periplasm by a predicted TonB-dependent transporter, SusC, where they are degraded to glucose by a neo-pullulanase/α-amylase, SusA, and an α-glucosidase/glucoamylase, SusB (37–41). SusR senses periplasmic maltose and upregulates *sus* transcription (42).

Some gut isolates lacking a pathway analogous to Sus encode an acarbose kinase and acarbose glucosidase that inactivate the drug (43, 44). Still, other enzymes from non-gut isolates can hydrolyze acarbose (45–47). The *Bacteroides* do not have homologs to these enzymes which made us question how or if they can circumvent acarbose-induced growth inhibition. Recent work demonstrated that some *Bacteroides* can take up fluorescently labeled maltodextrin in the presence of acarbose even while acarbose impairs growth (25). While this suggests acarbose can target an intracellular enzyme, this has not been shown mechanistically.

Bt and another model member of the Bacteroidota phylum, *Bacteroides ovatus* (Bo), have nearly identical *sus* loci (Fig. 1C). Strikingly, Bt growth on starch is inhibited by acarbose, but Bo is resistant to this growth inhibition. *In vitro* experiments demonstrate that growth on glucose and some non-starch substrates in acarbose is normal, strongly suggesting that one or more molecular features of Sus are responsible for the different phenotypes (48). The extracellular α-amylase is the least conserved protein between the two organisms with only 31% identity and 40% coverage (6) (Fig. 1C). Since acarbose inhibits glucoamylases, we initially hypothesized that BoSusG (BoGH13A_Sus_; Table 1) and SusG were differentially inhibited by acarbose. Via genetic manipulation, growth experiments, and inhibition kinetics of all Bo and Bt Sus enzymes, here, we show that although the primary target of acarbose in Bo and Bt is their respective periplasmic GH97 Sus enzyme, these enzymes do not explain the drastically different Bo and Bt acarbose phenotypes. To our surprise, Bo upregulates a non-Sus α-glucosidase gene (*bovatus_04772*), here termed BoGH97D, when exposed to maltose or acarbose. Bt encodes a nearly identical protein, BtGH97H, but its gene is not upregulated in conditions where acarbose impairs growth. Furthermore, constitutive expression of BoGH97D in Bt does not rescue its acarbose-induced lag phenotype. Instead, other non-enzymatic Sus features such as the TonB-dependent transporter and periplasmic transcriptional regulator may underpin Bo’s resistance to acarbose-induced growth inhibition.

**TABLE 1:** Bo Sus protein name abbreviations

Gene	Protein	Name used in text
*bovatus_03810*	BoSusR	BoSusR
*bovatus_03809*	BoGH13B_Sus_	BoSusA
*bovatus_03808*	BoGH97C_Sus_	BoSusB
*bovatus_03807*	BoSusC	BoSusC
*bovatus_03806*	BoSusD	BoSusD
*bovatus_03803*	BoGH13A_Sus_	BoSusG

Our work underscores the importance of interrogating the molecular interactions of acarbose with individual bacteria and the prominent role of GH97 enzymes in driving the observed acarbose phenotypes. Furthermore, we revealed that acarbose interferes not only with enzymatic starch breakdown but also with the ability of bacteria to transport and sense starch. Our data provide mechanistic insight into why members of the Bacteroidota may decrease in relative abundance in acarbose-treated individuals and influence how patients respond to acarbose treatment.

## RESULTS

### *Bacteroides thetaiotaomicron* is more sensitive to growth inhibition by acarbose than *Bacteroides ovatus*

*Bacteroides* growth inhibition by acarbose has been previously described for *Bacteroides thetaiotaomicron* (Bt), *Bacteroides fragilis* (Bf), *Phocaeicola dorei*, *Phocaeicola vulgatus,* and *Bacteroides xylanisolvens* (23–25). We extended these data and screened various Bacteroidota species for growth in both potato starch and amylopectin with or without 25 µM acarbose (Fig. 1D; Table 2). We chose two α-glucan polysaccharides for these experiments as some *Bacteroides* species grow better on one vs another. With a few exceptions, all species grew normally in glucose in the presence of 25 µM acarbose (Fig. S1 and S2). At least two replicates of all strains of a given species either grew or did n’t grow in both polysaccharides with acarbose after 144 h (Fig. S1 and S2). *Phocaeicola vulgatus* exhibited a mixed phenotype in which one strain grew in both polysaccharides in the presence of 25 µM acarbose, but two strains did not (Fig. 1D; Fig. S2). Within species that did grow, we observed a range of acarbose-induced ∆ lag times (defined as the difference in time to an OD_600_ of 0.3 in the acarbose vs untreated control) (Fig. 1E). Bf was inhibited by 8 µM acarbose, whereas *Bacteroides cellulosilyticus* (Bc) grew in amylopectin with 8 µM acarbose but was otherwise inhibited. *Bacteroides uniformis* (Bu) grew in 25 µM acarbose with a very extended lag compared to Bt and *Bacteroides ovatus* (Bo). Bf and Bu both have predicted LacI type regulators, major facilitator superfamily (MFS) transporters, and GH65s (maltose phosphorylases) in their predicted *sus* loci (of which there are two in Bu) but lack a SusR homolog. These features could make these species more susceptible to acarbose. However, Bc does not encode these traits in any of its four predicted *sus* loci, except for two predicted SusR homologs, and it is nearly as sensitive to acarbose as Bf (49). Given that Bo and Bt are both genetically tractable and have nearly identical *sus* loci, but exhibit different acarbose-induced lag times, we used these species as models to determine how acarbose inhibits bacterial growth.

**TABLE 2:** Structures of starch molecules referred to in text

Starch name	Structure		
Glucose (non-starch)	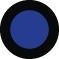		
Maltose (G2)	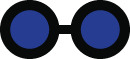		
Maltotriose (G3)	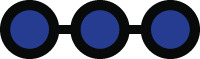		
Maltoheptaose (G7)	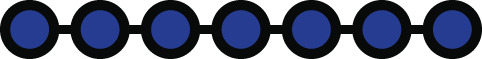		
Pullulan	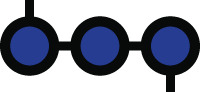 *n*		(50)
Glycogen	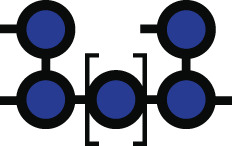 4–13		(51, 52)
Amylopectin	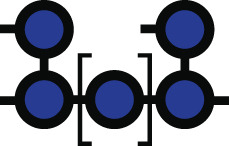 20–30		(53, 54)
Starch	Amylopectin (~80%)	Amylose (~20%)	
	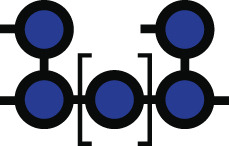 20–30	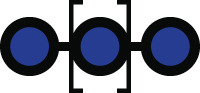 >1,000	(53, 55)

The Bt starch utilization system (Sus) is one model to study polysaccharide utilization loci (PUL) function in the Bacteroidota. Although BoSus has not been as extensively characterized, the organization of its *sus* locus is nearly identical to that of Bt (Fig. 1B and C). We were, therefore, surprised that Bo did not respond to acarbose similar to Bt (Fig. 1E and 2A). In a previous study, 10 µM acarbose led to nearly complete inhibition of Bt growth after 18 h on pullulan, a fungal polysaccharide comprised of repeating α1,6 linked maltotriose units, and potato starch (Table 2) (23). We repeated these experiments and extended the acarbose concentrations and polysaccharides tested.

To quantitatively assess the effects of acarbose on bacterial growth on a range of α1,4-linked substrates, we grew Bo and Bt in minimal media (MM) + 2.5 mg/mL glucose, maltose, maltoheptaose, glycogen, pullulan, amylopectin, and starch alone or with 10–500 µM acarbose (Table 2; Fig. 2B; Fig. S3A). From this, we calculated inhibitory acarbose concentrations for each polysaccharide which we defined as half the lowest acarbose dose that exhibited growth in at least two replicates by 72 h (Table 3). Both Bo and Bt tolerate up to 500 µM acarbose in all oligosaccharides tested: glucose, maltose, and maltoheptaose. When grown in pullulan, glycogen, amylopectin, or starch, Bt tolerates much less acarbose than Bo, except in starch (Table 3) (26). However, these inhibitory concentrations do not capture the striking differences in acarbose-induced ∆ lag times between Bo and Bt. At every concentration tested and with all tested α-glucans, Bt exhibited a significantly longer ∆ lag time than Bo (Fig. 2B; Fig. S3A).

**TABLE 3:** Inhibitory acarbose concentrations^*a*^

Aracarbose	Bo	Bt
Glucose	>250 µM	>250 µM
Maltose	>250 µM	>250 µM
Maltoheptaose	>250 µM	>250 µM
Glycogen	>250 µM	50 µM
Pullulan	125 µM	50 µM
Amylopectin	125 µM	37.5 µM
Starch	50 µM	50 µM

^*a*^ Half the lowest acarbose dose that afforded growth in at least two replicates by 72 h.

The difference in acarbose tolerance does not appear to be due to breakdown of the drug, as cell lysates of Bo and Bt grown on maltose with or without 50 µM acarbose cannot break down acarbose, even after extended incubation (Fig. S3B). Maltose induces *sus* expression, and the lysates are active against maltose and maltoheptaose. Furthermore, Bo and Bt cannot grow on acarbose as their sole carbon source (Fig. S3C). Therefore, we reasoned that acarbose likely differentially inhibits a Sus enzyme between the two organisms. To ascertain if acarbose targets the Sus machinery to elicit its inhibitory effects on Bt, and to a lesser extent on Bo, BoΔ*bovatus_03809–03803* and BtΔ*susABCDEFG*, hereafter referred to as BoΔSus and BtΔSus, respectively, were grown in the presence or absence of 50 µM acarbose. Note that both Bo∆Sus and Bt∆Sus cannot grow in minimal media with maltoheptaose or starch but do grow on maltose (Fig. 2C; Fig. S3D) (37). Although ΔSus strains lag on maltose compared to the respective wild-type strain, the addition of acarbose did not extend this lag or change growth kinetics (Fig. 2C and D). Likewise, no additional lag was observed for the ∆Sus strains grown in glucose and acarbose (Fig. S3E). These data led us to hypothesize that a difference in the Bo and Bt Sus was responsible for Bo’s resistance to acarbose-induced growth inhibition.

### Sus outer membrane α-amylases are not the source of the different Bo and Bt acarbose phenotypes

**Fig 3:**
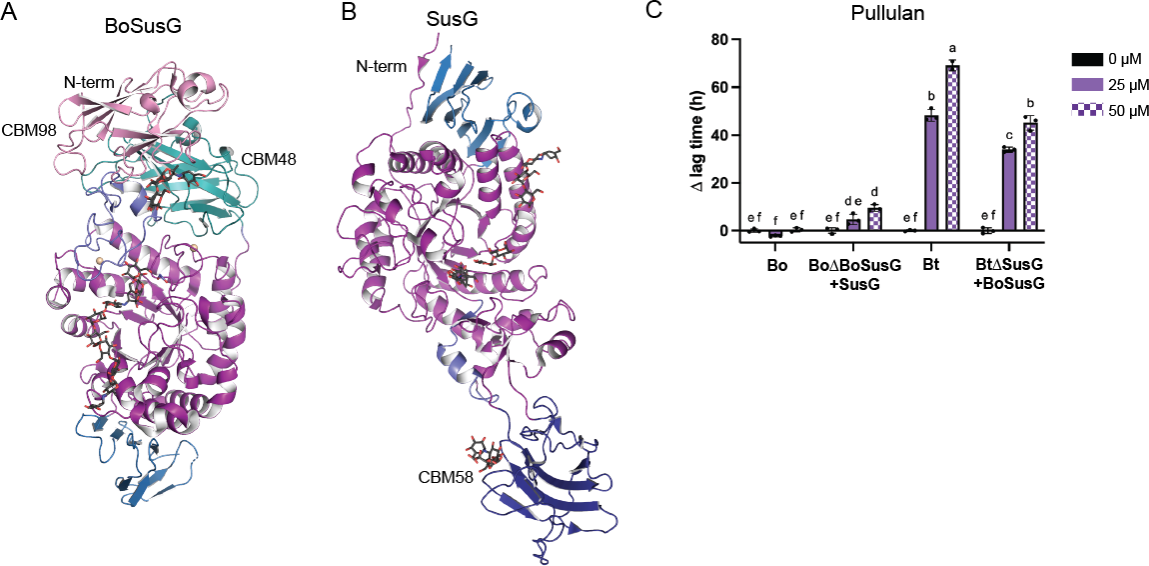
The extracellular amylases BoSusG and SusG are not the source of the different acarbose phenotypes in Bo and Bt, respectively. (**A**) The crystal structure of BoSusG bound to acarbose (PDB: 8DL2 [6]). The GH13 A domain is in purple, B domain in slate, and C domain in blue. CBM98 is pink and CBM48 is teal. (**B**) The crystal structure of SusG bound to acarbose (PDB: 3K8M [36]). The GH13 domains are colored as in A. CBM58 is dark blue. They are oriented with their N-termini at the top of the figure, as both are anchored to the outer membrane via lipidation of an N-terminal Cys. Figures were rendered in PyMOL (57). (C) Given that BoSusG does not optimally complement Bt∆SusG, these growths were performed with bacteria pre-grown on minimal media (MM) + 5 mg/mL maltose to induce *sus* expression and then inoculated into MM + 2.5 mg/mL pullulan in the indicated acarbose concentrations. The difference in time to OD_600_ of 0.3 between the treated and untreated conditions (∆ lag time) is graphed. Statistical analyses were performed using a two-way ANOVA using a cutoff of *P* ≤ 0.05. Conditions with the same letter(s) were not significantly different from one another. All growths were performed in triplicate. The mean and standard deviation are shown.

The most likely candidate for differential inhibition is the extracellular α-amylase enzymes, as this is the most obviously different component of the loci (Fig. 1C). BoGH13A_Sus_ (hereafter referred to as BoSusG; Table 1) and SusG are both α-amylases belonging to the glycoside hydrolase family 13 (GH13). BoSusG belongs to subfamily 47, whereas SusG belongs to subfamily 36. Furthermore, they have vastly different structural properties (6, 36) (Fig. 3A and B). BoSusG has two N-terminal carbohydrate-binding modules (CBMs): CBM98, which binds maltooligosaccharides, and CBM48, which does not (Fig. 3A) (6). SusG features a CBM58 that interrupts its catalytic domain (Fig. 3B) (36). Both enzymes have A, B, and C domains that are GH13 hallmarks although the BoSusG B domain is much smaller than SusG’s (56). BoSusG and SusG have signal peptides that destine them to the outer leaflet of the outer membrane via lipidation at an N-terminal cysteine residue. The orientation of the SusG GH13 domain, however, is opposite that of BoSusG relative to the outer membrane (Fig. 3A and B). Given the extensive differences between BoSusG and SusG at a structural level, we hypothesized that SusG would be more inhibited by acarbose than BoSusG in congruence with the respective organism’s acarbose growth phenotype.

To quantify acarbose inhibition kinetics of each enzyme, IC_50_ values were determined using a fluorescent starch assay. To our surprise, BoSusG was more inhibited by acarbose than SusG, borne out by a 10-fold lower IC_50_ (Table 4; Fig. S4A and B). The SusG IC_50_ is in rough alignment with the growth-derived inhibitory acarbose concentrations for polysaccharides in Table 3, but the IC_50_ for BoSusG is much lower than acarbose concentrations tolerated by Bo during growth. In previous work, we showed that SusG displays a minimal amount of acarbose hydrolysis *in vitro* over time using elevated enzyme concentrations, but BoSusG cannot break down acarbose (6, 36).

**TABLE 4:** Enzyme kinetic parameters

Protein	*K*_*M*_ (mM)	*k*_cat_ (s^−1^)	*k*_cat_/*K*_*M*_ (s^−1^ mM^−1^)	Inhibition constant
BoSusG	N/A^*d*^	N/A^*d*^	N/A^*d*^	2.2 ± 0.35 µM^*a*^
SusG	N/A^*d*^	N/A^*d*^	N/A^*d*^	68.2 ± 13.8 µM^*a*^
BoSusA	0.135 ± 0.010	18.2 ± 0.5	135	122.6 ± 9.2 nM^*b*^
SusA	0.117 ± 0.009	13.8 ± 0.3	120	94.7 ± 6.9 nM^*b*^
BoSusB	0.078 ± 0.015	21.5 ± 1.2	276	69.3 ± 8.9 nM^*c*^
SusB	0.089 ± 0.013	23.9 ± 1.1	269	53.5 ± 12.5 nM^*c*^
BoGH97D	0.088 ± 0.015	51.2 ± 2.8	582	133.8 ± 22.4 nM^*c*^
BtGH97H	0.088 ± 0.014	48.6 ± 2.5	552	162.4 ± 24.3 nM^*c*^

^*a*^ IC_50_.

^*b*^ α*K*_*i*_.

^*c*^ *K*_*i*_.

^*d*^ N/A = not applicable. These values were not calculated as part of this work.

To substantiate our *in vitro* findings, the extracellular α-amylase genes were swapped between Bo and Bt in their respective *sus* loci to test whether the acarbose phenotype tracked with the expressed enzyme. BoΔBoSusG and BtΔSusG cannot grow on starch polysaccharides (6, 30, 34). We have previously demonstrated that *susG* complements a BoΔBoSusG strain on potato starch, potato amylopectin, pullulan, and glycogen (6). However, *bovatus_03803* (the gene for BoSusG) poorly complements BtΔ*susG* growth on potato amylopectin and glycogen but restores growth on pullulan and, to a lesser extent, potato starch when cells are pre-grown on maltose to induce *sus* expression (6). Therefore, acarbose-induced growth lags on pullulan in the BoΔBoSusG + SusG and BtΔSusG + BoSusG strains were assessed. Both strains grew normally in glucose (6) (Fig. S4C). BoSusG imparted a small benefit to Bt∆SusG compared to Bt and BoΔBoSusG + SusG lagged slightly more compared to Bo, both with statistical significance. However, the swapped strains nonetheless responded to acarbose more like the parent strain and without changing the overall phenotype (Fig. 3C). Together with the inhibition data, this suggests that the different Bo and Bt responses to acarbose are not primarily driven by BoSusG or SusG.

### Acarbose likely competes with maltooligosaccharides for transport through BoSusC and SusC

**Fig 4:**
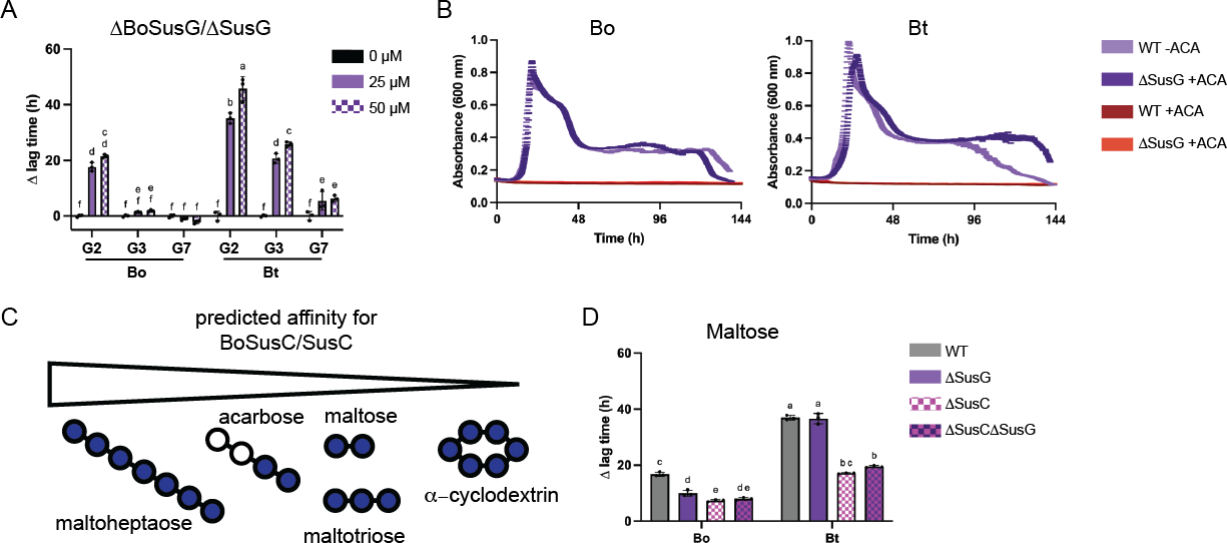
Acarbose likely competes with maltooligosaccharides for transport through BoSusC and SusC. (**A**) Bacteria were pre-grown in minimal media (MM) with glucose and back diluted in MM + 2.5 mg/mL maltose (G2), maltotriose (G3), or maltoheptaose (G7) with and without the indicated acarbose concentrations. The difference in time to OD_600_ of 0.3 between the treated and untreated conditions (∆ lag time) is graphed. (**B**) Bacteria were grown as described in Fig. 2B in 2.5 mg/mL α-cyclodextrin with or without 50 µM acarbose. (**C**) Based on panels A and B, we predict that maltoheptaose has the highest affinity for BoSusC/SusC, whereas αCD has the lowest. (**D**) Bacteria were pre-grown in MM and back diluted in MM + 2.5 mg/mL maltose with or without 50 µM acarbose. The difference in time to OD_600_ of 0.3 between the treated and untreated conditions (∆ lag time) is graphed. Statistical analyses in panels A and D were performed with a two-way ANOVA. Conditions with the same letter(s) were not significantly different from one another. A cutoff of *P* ≤ 0.05 was used. All growths were performed in triplicate. The mean and standard deviation are shown in the ∆ lag graphs, while the growth curves show the average of all three growths.

BoΔBoSusG and BtΔSusG can grow on short maltooligosaccharides (6, 30, 34, 35). Therefore, to examine acarbose-induced growth inhibition in the absence of BoSusG and SusG, we grew the deletion mutants on various maltooligosaccharides with or without acarbose. In the absence of BoSusG or SusG, acarbose inhibited growth in a size-dependent manner with growth on shorter maltooligosaccharides displaying a longer lag time (Fig. 4A). Acarbose did not affect growth on glucose (Fig. S5A). Furthermore, the shape of the oligosaccharide influenced growth. Both Bo and Bt grew on α-cyclodextrin (αCD) independent of BoSusG and SusG, respectively. However, WT and ∆SusG strains of Bo and Bt did not grow on αCD in the presence of acarbose (Fig. 4B). Taken together, these results suggest that (i) acarbose may compete with maltooligosaccharides for transport through BoSusC and SusC in a size and shape-dependent manner (Fig. 4C) and (ii) acarbose can enter the periplasm to inhibit Sus periplasmic enzymes.

To probe the first possibility, strains lacking BoSusC/SusC were grown in maltose with or without acarbose since growth on maltose, but not longer maltooligosaccharides, does not require SusC in Bt (Fig. S5B) (34, 37). In the mutants lacking this transporter, cells lagged significantly less in the presence of maltose plus acarbose compared to the WT cells (Fig. 4D; Fig. S5C and D). We hypothesize that this reduced lag is because maltose transport is BoSusC/SusC independent, whereas acarbose transport, due to its larger size, is SusC/BoSusC dependent. ∆BoSusC and ∆SusC strains exhibited a growth benefit in glucose with acarbose compared to the WT strains (Fig. S5E). Even though Bo and Bt are not affected by acarbose when grown in glucose, there may be some benefit to keeping acarbose outside of the cell in the strains lacking an outer membrane Sus transporter. Finally, discrepant Bo and Bt acarbose phenotypes were still observed in the absence of the BoSusC/SusC transporter and extracellular amylase (Fig. 4D). Together, these data supported that the target of acarbose inhibition is intracellular, as deletion of the BoSusC/SusC transporters provided partial relief of inhibition, and the surface amylases do not drive the difference in phenotype between Bo and Bt.

### Periplasmic GH13 enzymes are not the source of the different Bo and Bt acarbose phenotypes

**Fig 5:**
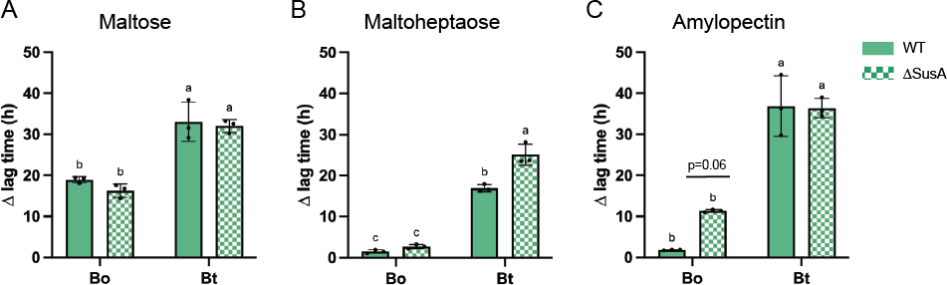
Periplasmic Sus GH13 enzymes do not underpin the Bo and Bt responses to acarbose. (**A–C**) Bo and Bt were pre-grown in minimal media (MM) with glucose and back diluted into MM + 2.5 mg/mL maltose, maltoheptaose, or amylopectin with or without 50 µM acarbose. The difference in time to OD_600_ of 0.3 between the treated and untreated conditions (∆ lag time) is graphed. Statistical analyses were performed with a two-way ANOVA. Conditions with the same letter(s) were not significantly different from one another. A cutoff of *P* ≤ 0.05 was used. All growths were performed in triplicate. The mean and standard deviation are shown.

Because deletion of the *sus* locus relieves the acarbose-induced growth lag in MM plus maltose, we next examined the influence of the periplasmic Sus enzymes in mediating sensitivity to the drug. The genes encoding BoGH97C_Sus_ (hereafter referred to as BoSusB; Table 1) and SusB were mutated to introduce a stop codon after eight amino acids, as an in-frame deletion of *susB* has polar effects in Bt (38). While *susA* is transcribed separately from *susBCDEFG* (38), genes for BoGH13B_Sus_ (hereafter referred to as BoSusA; Table 1) and SusA were also knocked out via an early stop codon for consistency. Bo∆BoSusA and Bt∆SusA grew normally in glucose with acarbose (Fig. S6A). Given that the deletions grew in both short and long starch molecules, their acarbose induced ∆ lag phenotypes were assessed in maltose, maltoheptaose, and amylopectin. BtΔSusA still exhibited an acarbose-induced ∆ lag in all three carbon sources (38, 58) (Fig. 5A through C). Conversely, Bo demonstrated a modest ∆ lag in maltose and a very small ∆ lag in 50 µM acarbose when grown on maltoheptaose or amylopectin. Deleting BoSusA rendered the organism slightly more susceptible to acarbose although the difference was not statistically significant, rather than relieving the already miniscule growth lag (Fig. 5A through C).

To corroborate that BoSusA and SusA are not the primary drivers of the different acarbose responses, the enzymes were recombinantly expressed in *Escherichia coli* and purified to compare their activities. SusA is known to target α1,4 linkages in maltose through maltoheptaose, β-cyclodextrin (βCD), and pullulan (producing panose as a product) and does not recognize α1,6 bonds (39) (Fig. S6B). Substrate preferences of BoSusA were qualitatively compared to those in SusA to determine if BoSusA has activity not found in SusA that would explain the Bo acarbose phenotype. Activities were compared by overnight incubation with various substrates and analysis via thin-layer chromatography. Both proteins exclusively targeted the α1,4 linkages in starch oligosaccharides as panose, isomaltose, and dextran (polymer of α1,6-linked glucose) were not broken down (Fig. S6B through D). BoSusA and SusA are annotated as neopullulanases with cyclomaltodextrinase N- and C-terminal domains and belong to the recently identified GH13_46 subfamily (59). Both enzymes broke down pullulan (into panose), αCD, βCD, the pullulan oligosaccharides 6^3^-α-d-glucosyl-maltotriose (GM), 6^3^-α-d-glucosyl-maltotriosyl-maltotriose (GMM), and amylopectin (Fig. S6B through D). Neither was active on glycogen and BoSusA was more active than SusA on potato starch. Most importantly, BoSusA and SusA did not break down acarbose (Fig. S6C and D).

BoSusA and SusA inhibition by acarbose was quantified using pNP-maltohexaose (pNP-G6) as a substrate, for which the enzymes have comparable catalytic efficiency (Table 4; Fig. S7A and B). To our surprise, acarbose modeled best as an uncompetitive inhibitor of both enzymes, implying that acarbose only binds to the enzyme-substrate complexes and not to free enzyme (Fig. S7C and D). This was unexpected since acarbose is typically a mixed or a competitive enzyme inhibitor (60, 61). Even though our kinetic data were best modeled via uncompetitive inhibition, we are inclined to conclude that acarbose is instead a mixed inhibitor of these enzymes because it binds to free enzyme as measured by isothermal titration calorimetry (Fig. S8A through D). Wild-type enzyme has two acarbose-binding sites, whereas a catalytically inert variant of each enzyme (D331N) has one (Table S1). Both BoSusA and SusA are dimers (Fig. S8E), raising the possibility of allosteric modulation. Nonetheless, the inhibition constants are the same order of magnitude (α*K*_*i*_s of 123 vs 95 nM for Bo and Bt enzymes, respectively, (Table 4), suggesting that BoSusA and SusA do not explain the Bo and Bt responses to acarbose.

### Periplasmic Sus GH97s drive acarbose responses but do not underpin the different Bo and Bt phenotypes

**Fig 6:**
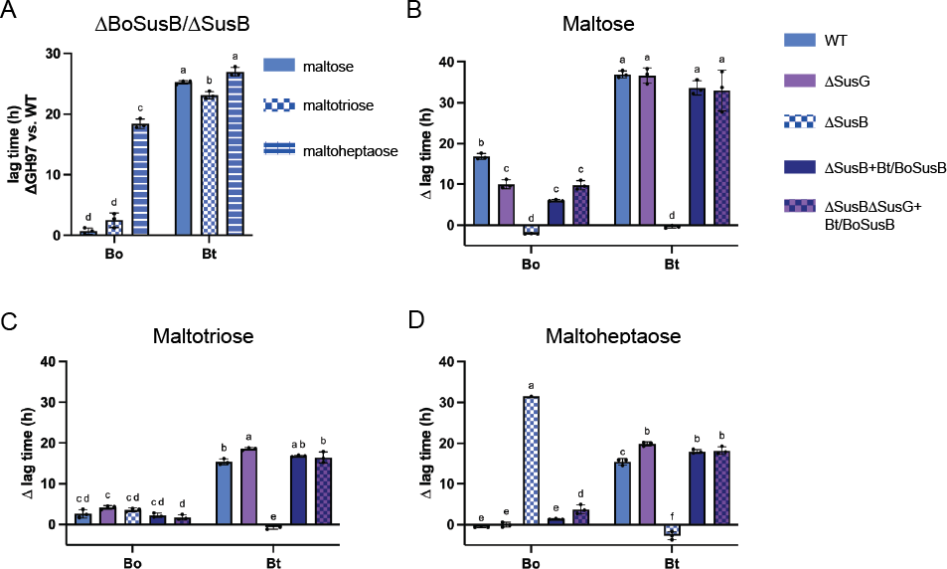
Periplasmic Sus GH97 enzymes are the primary acarbose target but do not explain the different Bo and Bt phenotypes in acarbose. (**A**) Bacteria were grown in minimal media (MM) with glucose and back diluted into MM + 2.5 mg/mL maltose, maltotriose, or maltoheptaose. Difference in time to OD_600_ of 0.3 between WT Bo and Bo∆BosusB and between WT Bt and Bt∆SusB is shown. (**B–D**) Bacteria were pre-grown in MM with glucose and back diluted into MM + 2.5 mg/mL maltose, maltotriose, or maltoheptaose with or without 50 µM acarbose. ∆SusG corresponds to Bo∆BoSusG and Bt∆SusG. ∆SusB corresponds to Bo∆BoSusB and Bt∆SusB. ∆SusG∆SusB + Bt/BoSusB and ∆SusB + Bt/BoSusB correspond to the SusB swapped strains. The difference in time to OD_600_ of 0.3 between the treated and untreated conditions (∆ lag time) is graphed. Statistical analyses were performed with a two-way ANOVA. Conditions with the same letter(s) were not significantly different from one another. A cutoff of *P* ≤ 0.05 was used. All growths were performed in triplicate. The mean and standard deviation are shown, except in panel D. Only one replicate of Bo∆BoSusB grew in maltoheptaose.

Unlike the SusA knockouts, the deletion of the genes encoding the SusBs (BoΔBoSusB and BtΔSusB) eliminated growth on amylopectin (Fig. S9A and B). Our results with Bt differed on this point from previous work by the Salyers lab but are in accordance with data collected from barcoded transposon libraries of Bt (38, 58, 62). Given that the deletion mutants cannot grow on polysaccharide, they were screened in maltose, maltotriose, and maltoheptaose (Table 2). Without acarbose, Bt∆SusB exhibited a significant growth lag compared to Bt on maltose, maltotriose, and maltoheptaose, while Bo∆BoSusB lagged on maltoheptaose compared to Bo but not maltose or maltotriose (Fig. 6A). BtΔSusB and BoΔBoSusB were nonetheless screened for acarbose-induced growth inhibition in all three oligosaccharides. Neither deletion strain exhibited an acarbose-induced ∆ lag when grown in glucose (Fig. S9C). In the absence of BoSusB and SusB, Bo and Bt no longer had an acarbose-induced ∆ lag in maltose (Fig. 6B; Fig. S9D). Compared to WT, the acarbose-induced ∆ lag in maltotriose was rescued in Bt∆SusB, but Bo∆BoSusB grew similar to WT Bo but had a slight kinetic growth defect without acarbose that was not exacerbated by the drug (Fig. 6C; Fig. S9E). Strikingly, when grown on maltoheptaose, Bt∆SusB did not have a growth defect, but Bo∆BoSusB growth was impaired by acarbose (Fig. 6D; Fig. S9F). To further examine, we swapped these genes between organisms in the context of their *sus* loci in hopes of swapping acarbose phenotypes. Swapped strains were grown on the same three oligosaccharides for comparison to the ∆SusB strains. BtΔSusB + BoSusB and BoΔBoSusB + SusB grew like their wild-type counterparts with or without the outer membrane SusGs which are active on maltoheptaose (BoSusG) and maltoheptaose and maltotriose (SusG) (Fig. 6B through D; Fig. S9G through I) (6, 36). BoΔBoSusB + SusB and Bo∆BoSusG∆BoSusB + SusB had a slightly smaller ∆ lag compared to WT Bo when grown in maltose, but not maltotriose or maltoheptaose (Fig. 6B through D; Fig. S9G through I).

Furthermore, BoSusB and SusB have similar substrate preferences. Our work here, and previously published work on SusB, demonstrate that both enzymes are exo-acting glucoamylases that accommodate α1,6 and α1,4 linkages and do not break down acarbose (39, 40) (Fig. S10A).

BoSusB and SusB kinetic parameters using pNP-glucose as a substrate demonstrated they have similar catalytic efficiencies and the SusB *K*_*i*_ (54 nM) for acarbose was comparable to published work (150 nM and 108 nM) (Table 4; Fig. S10B through E) (40, 41). Remarkably, BoSusB had a similar *K*_*i*_ (69 nM). SusB has been structurally characterized and is known to be competitively inhibited by acarbose (40, 41). We solved a co-crystal structure of BoSusB with acarbose bound in the active site near the dimer interface (Table S2; Fig. S10F). Acarbose binds identically to the SusB and BoSusB active sites (Fig. S10G). Collectively, these data suggest that although BoSusB and SusB drive most of the acarbose phenotype in Bo and Bt, respectively, they are not responsible for the acarbose-induced phenotypic differences.

### Bo expresses a GH97 enzyme whose homolog is not expressed by Bt

Because Bo and Bt ΔSus strains no longer exhibit a growth lag due to acarbose when grown in maltose (Fig. 2C), we had hypothesized that differences in Sus were responsible for the phenotypic growth differences between Bo and Bt in the presence of acarbose. However, the *sus-*encoded enzymes of both organisms have nearly identical responses to acarbose and swapping these components does not convert the phenotypes. Thus, we concluded that while SusB is a significant target of acarbose for both organisms, we hypothesized that Bo has an additional mechanism to subvert acarbose growth inhibition for two reasons. First, in the absence of acarbose, BoΔSus consistently grows up faster than BtΔSus in maltose, suggesting it might harbor a glucosidase/glucoamylase not found in Bt (Fig. 2D). Second, without acarbose, Bo∆BoSusB did not exhibit a growth lag on maltose or maltotriose compared to WT Bo but Bt∆SusB did (Fig. 6A).

**Fig 7:**
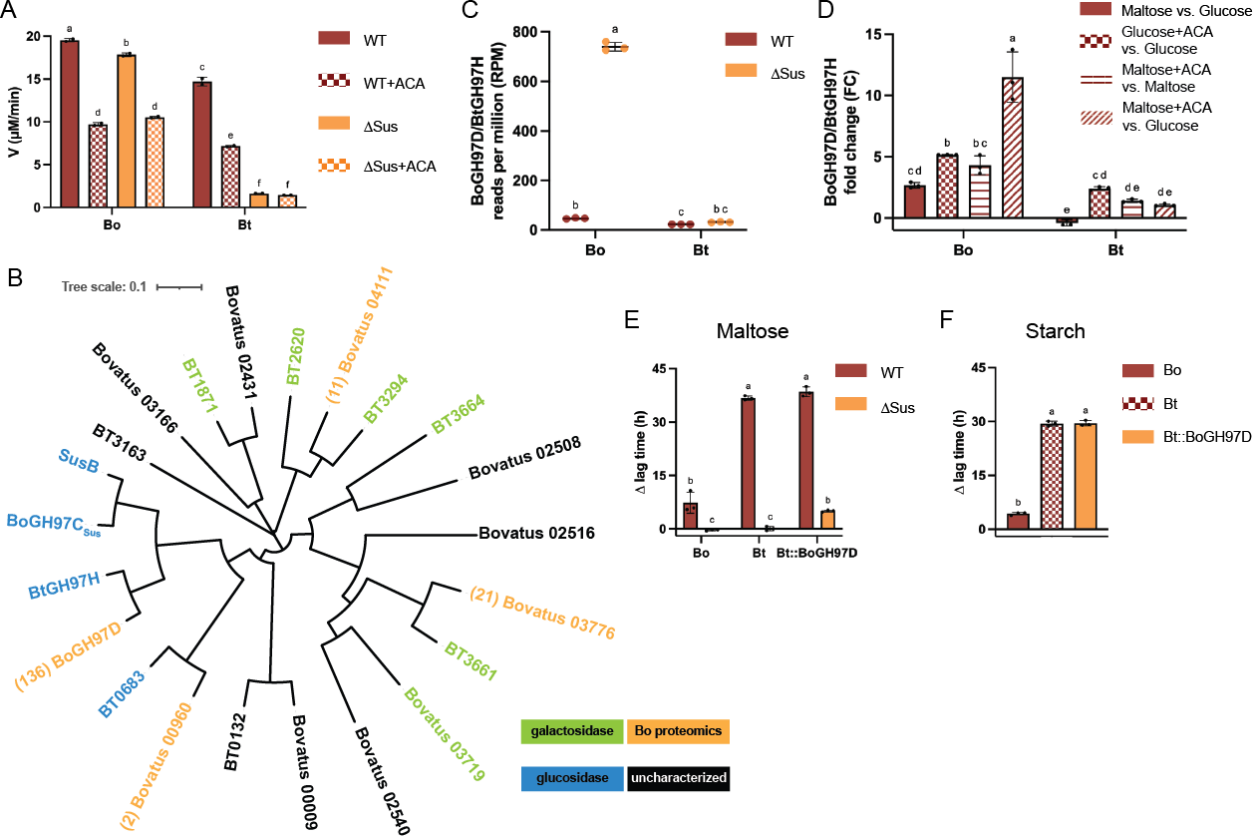
Bo upregulates a non-Sus GH97, BoGH97D, that does not rescue the acarbose-induced growth lag when expressed by Bt. (**A**) WT and ∆Sus strains of Bo and Bt were grown in minimal media (MM) + 5 mg/mL maltose to the same OD_600_ and pelleted. Pellets were washed in PBS, and cells were sonicated to release contents. Lysates were assayed in 1 mM pNP-Glc with or without 1 µM acarbose in duplicate. (**B**) Bo∆Sus cellular contents following growth in maltose were purified according to the methods to increase specific activity on pNP-Glc. Proteins in the purest and most active fractions were identified via LC-MS/MS. The most abundant predicted α-glucan active enzyme was *bovatus_04772* (BoGH97D). A phylogenetic tree of all Bo and Bt GH97s is shown along with the number of peptide to spectrum (PSM) matches observed in the proteomics experiment. Locus tags with colored names have previously been characterized biochemically (38, 40, 41, 63–68) or were characterized for this study (BoGH97D, BtGH97H). (**C**) WT and ∆Sus strains of Bt and Bo were grown in triplicate in MM + 5 mg/mL maltose. Total RNA was purified and subjected to RNAseq. Reads per million are displayed. (**D**) Bo and Bt were grown in triplicate in MM with 5 mg/mL glucose or maltose with or without 50 µM acarbose and total RNA was purified. qPCR was performed to determine the fold change in BoGH7D and BtGH97H expression in the following conditions: maltose vs glucose; glucose + 50 µM acarbose (ACA) vs glucose; maltose + 50 µM acarbose (ACA) vs maltose; maltose + 50 µM acarbose (ACA) vs glucose. (**E, F**) The indicated strains were pre-grown on MM + 5 mg/mL maltose to recapitulate growths used for RNAseq and otherwise grown in MM + 2.5 mg/mL maltose or starch with or without 50 µM acarbose. The difference in time to OD_600_ of 0.3 between the treated and untreated conditions (∆ lag time) is graphed. All statistical analyses were performed with a two-way ANOVA. Conditions with the same letter(s) were not significantly different from one another. A cutoff of *P* ≤ 0.05 was used. All growths were performed in triplicate. The mean and standard deviation are shown.

To test our hypothesis, WT and ΔSus strains of Bo and Bt were grown in maltose and harvested at similar ODs. Maltose was used because it is the smallest starch molecule that ∆Sus strains can grow on. Bacteria were pelleted, sonicated, and centrifuged to clarify debris. The clarified lysates were assayed for pNP-glucose activity using 1 mM substrate. As expected, wild-type lysates from both organisms exhibited robust pNP-glucose activity due to BoSusB and SusB (Fig. 7A). BtΔSus recapitulated published work in a BtΔSusB strain and had minimal pNP-glucose activity (38). BoΔSus, however, had activity comparable to WT Bo, suggesting it harbors one or more non-Sus backup glucosidases/glucoamylases (Fig. 7A). To our surprise, this activity was inhibited by 1 µM acarbose in the conditions tested (Fig. 7A).

We used two complementary approaches to identify Bo glucosidases. First, activity-guided fractionation was performed from BoΔSus grown in maltose since it cannot grow on larger starch molecules. Following sonication and centrifugation, clarified lysates were subjected to ammonium sulfate cuts and chromatographic separation. Throughout, fractions were tested for pNP-glucose activity and the identities of proteins in the purest and most active fractions were determined via LC MS/MS (Table S3). The most abundant enzyme that we predicted would exhibit pNP-glucose activity was *Bovatus_04772* (hereafter referred to as BoGH97D) annotated as a GH97. Of other glycoside hydrolase families with α-glucan activity (GH13, GH15, GH31, GH57, and GH77), two GH31s were identified by a single peptide for each (49). Bo does not encode any other predicted α-glucan active GHs such as maltose phosphorylases (GH65), GH4, GH63, or GH122s in its genome (49). Furthermore, of the four GH97s identified by proteomics, two are predicted α-galactosidases and the other predicted α-glucosidase had a single peptide sequenced (Fig. 7B; Table S3). Bt, however, encodes a homolog of BoGH97D, Bt4581 (hereafter referred to as BtGH97H), which is 90% identical.

We also performed RNAseq of WT and ∆Sus strains of Bo and Bt grown in maltose since ∆Sus strains cannot grow on longer starch molecules. The BoGH97D transcript was nearly 16-fold more abundant in Bo∆Sus compared to WT Bo, whereas the BtGH97H transcript was only 1.5-fold more abundant in Bt∆Sus compared to WT Bt (Fig. 7C; Table S4). Furthermore, no Bt transcripts for GH31s or GH97s passed our significance or fold-change threshold (*P* ≤ 0.05; twofold change; Table S5). While *Bovatus_02431*, a GH97 with predicted α-galactosidase activity based on phylogeny, did exceed our threshold, there was one-tenth as much transcript as BoGH97D, and it was not identified by proteomics (Fig. 7B; Tables S3 and S4). A single Bo GH31, *Bovatus_03177*, also passed our cutoff but is 40 times less abundant than BoGH97D and was not identified by proteomics (Tables S3 and S4).

BoGH97D and BtGH97H were purified and confirmed to be α-glucosidases and not α-galactosidases (Fig. S11A). They break down all α1,4 and α1,6 linked substrates that BoSusB and SusB are active on (Fig. S11B). BoGH97D and BtGH97H are predicted to localize to the periplasm due to their SPI signal peptides (69). Based on comparisons to known determinants of inverting (vs retaining) activity in SusB, we predict BoSusB, BoGH97D, and BtGH97H to be inverting (Fig. S11C) (41). BoGH97D and BtGH97H had comparable catalytic efficiencies using pNP-Glc as a substrate, and both enzymes are competitively inhibited by acarbose with comparable *K_i_*s (134 vs 162 nM for BoGH97D and BtGH97H, respectively) (Table 4; Fig. S12A through D).

Since both BoGH97D and BtGH97H are active enzymes, we reasoned that if BoGH97D is at least partly responsible for the Bo acarbose phenotype, it is regulated differently than BtGH97H. Via qPCR, we determined that BoGH97D was upregulated by maltose alone and acarbose compared to glucose alone. The effect was additive as the greatest upregulation was observed in maltose plus acarbose vs glucose alone (Fig. 7D). Conversely, BtGH97H was not upregulated by maltose but was upregulated by acarbose compared to glucose alone. However, acarbose upregulated BoGH97D over two times as much as BtGH97H when bacteria were grown in glucose. When grown in maltose, BtGH97H was not upregulated by the addition of acarbose, but BoGH97D was (Fig. 7D). These data suggest that BoGH97D, but not BtGH97H, is upregulated by acarbose in the context of growth on starch.

### BoGH97D does not confer acarbose resistance to Bt

Our attempts to delete BoGH97D from the Bo genome were unsuccessful, so we could not assess its contribution to acarbose resistance. Instead, we overexpressed BoGH97D constitutively in Bt and Bt∆Sus to see if it rescued part of the acarbose induced ∆ lag (70). First, we repeated the lysate experiments and showed that Bt∆Sus::BoGH97D lysates had more pNP-Glc activity than Bt∆Sus ones, but not as much as Bo∆Sus lysates (Fig. S13A). Bt::BoGH97D lysates did not have more glucosidase activity than Bt lysates, most likely due to the amount of SusB in the WT lysates. Nonetheless, we screened these Bt strains for resistance to acarbose. Bt::BoGH97D and Bt∆Sus::BoGH97D grew similar to Bt and Bt∆Sus in the absence of acarbose when grown in maltose or starch (Fig. S13B and C). The longest usable carbon source for each strain was used: maltose for Bt∆Sus::BoGH97D and starch for Bt::BoGH97D. Neither strain exhibited an acarbose-induced ∆ lag in glucose (Fig. S13D). Bt∆Sus::BoGH97D grew similar to Bt∆Sus in maltose, and Bt::BoGH97D grew similar to Bt in maltose and starch (Fig. 7E and F). Thus, BoGH97D did not provide Bt any growth advantage in the presence of acarbose, suggesting that differential regulation of the non-Sus enzymes BoGH97D and BtGH97H does not contribute significantly to the different acarbose phenotypes between Bo and Bt.

### Acarbose and maltose upregulate BoSus to a greater extent than BtSus

**Fig 8:**
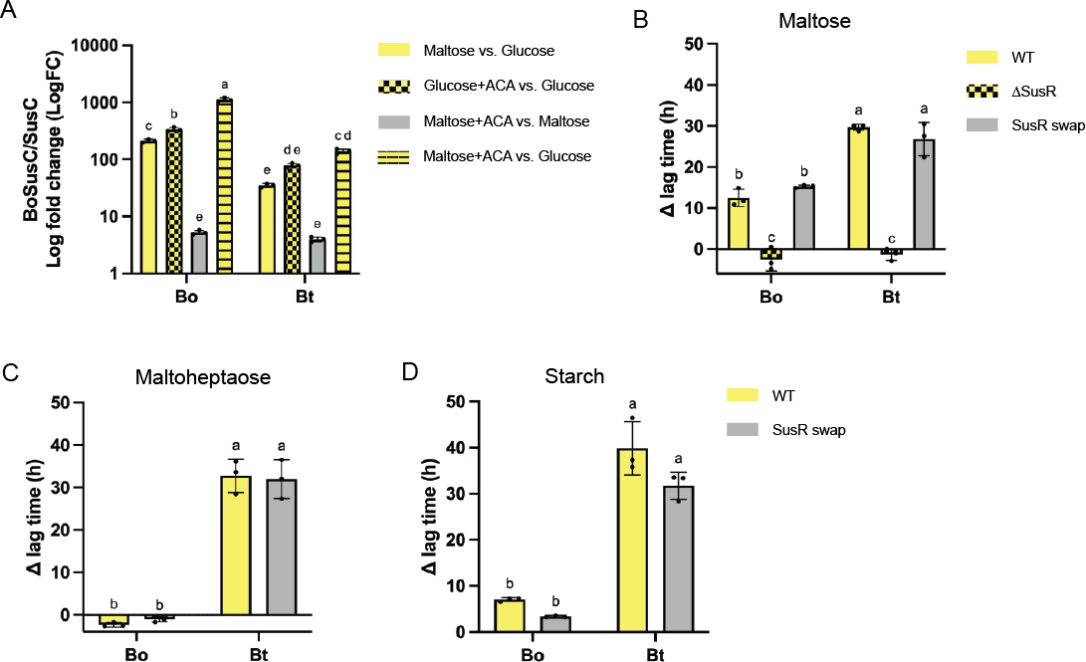
Although acarbose targets BoSusR and SusR, the regulators are not the source of the acarbose ∆ lag phenotype. (**A**) Bo and Bt were grown in triplicate in 5 mg/mL glucose or maltose with or without 50 µM acarbose, and total RNA was purified. qPCR was performed to determine the fold change in BoSusC and SusC expression in the following conditions: maltose vs glucose; glucose + 50 µM acarbose (ACA) vs glucose; maltose + 50 µM acarbose (ACA) vs maltose; maltose + 50 µM acarbose (ACA) vs glucose. Statistical analyses were performed with a two-way ANOVA. Conditions with the same letter(s) were not significantly different from one another. A cutoff of *P* ≤ 0.05 was used. Note that due to the magnitude of some of the responses, Log fold change is displayed. The mean and standard deviation are shown. (B–D) WT, ∆Sus, and SusR swap strains of Bo and Bt were pre-grown on minimal media (MM) with glucose and back diluted into MM + 2.5 mg/ml maltose, maltoheptaose, or starch with our without 50 µM acarbose. The difference in time to OD_600_ of 0.3 between the treated and untreated conditions (∆ lag time) is graphed. All statistical analyses were performed with a two-way ANOVA. Conditions with the same letter(s) were not significantly different from one another. A cutoff of *P* ≤ 0.05 was used. All growths were performed in triplicate. The mean and standard deviation are shown.

An additional periplasmic component we have not addressed is the regulator BoSusR/SusR. We wondered if Bo and Bt upregulate their *sus* loci differently in response to maltose and acarbose. To test this, qPCR on the genes for BoSusC and SusC was performed. To our surprise, compared to glucose alone, acarbose and glucose upregulated *sus* more than maltose in both organisms. Fifty micromolar acarbose was used in these experiments, whereas the bacteria were grown in 5 mg/mL maltose (~14.6 mM). Furthermore, the magnitude of responses between Bo and Bt was strikingly different as maltose upregulated BoSus ~210-fold and BtSus ~35-fold. The addition of 50 µM acarbose to the glucose culture upregulated BoSus ~335-fold and BtSus ~80-fold. On the other hand, the addition of 50 µM acarbose to cultures grown in maltose only upregulated Bo and BtSus ~5-fold compared to maltose alone. The combination of maltose and acarbose compared to glucose most potently upregulated Sus in both organisms (Fig. 8A). At the transcriptional level, BoSusR and SusR levels were comparable between all conditions in Bo and Bt, respectively, as has been previously observed in Bt (Fig. S14A) (42). BoSusR was slightly more upregulated in maltose and acarbose vs maltose alone (~1.3-fold) compared to SusR (~0.75-fold).

Given how robustly maltose and acarbose upregulated BoSus compared to BtSus, we investigated the contribution of BoSusR and SusR to the acarbose phenotype in each organism. Bo∆BoSusR and Bt∆SusR cannot grow on maltoheptaose or starch and growth on glucose is not inhibited by acarbose (Fig. S14 B and C) (71). Bo∆BoSusR and Bt∆SusR growth on maltose is not inhibited by acarbose, likely because there is less or no SusB produced (Fig. 8B; Fig. S14D). However, this is different than the Δ*sus* strains, which are *susA-G* deletions in both species and do include *susR*. Bo∆Sus grew up faster in maltose than Bt∆Sus (Fig. 2C), whereas Bo∆BoSusR and Bt∆SusR grew similarly in the same substrate (Fig. S14D). Although deleting the SusRs relieved the acarbose ∆ lag phenotype in both organisms grown in maltose, swapping SusR genes between each organism did not swap the phenotype when strains were grown in maltose, maltoheptaose, or starch (Fig. 8B through D). All three carbohydrates were tested since SusR likely senses and responds to maltooligosaccharides besides maltose.

## DISCUSSION

Acarbose is an effective type 2 diabetes medication that is showing promise for potential anti-aging and cardioprotective effects. Acarbose exposure also affects the gut microbiome. Among these effects is a reduction in the relative abundance of *Bacteroides* species, including *Bacteroides thetaiotaomicron* (Bt), *Phocaeicola dorei*, and *Phocaeicola vulgatus* (13). Acarbose impairs the growth of all three species when using starch *in vitro* as a carbon source (23, 24) (Fig. 1D). Here, we fill in some of the mechanistic gaps between exposure to acarbose and changes in the gut microbiome and host. Given that *Bacteroides ovatus* (Bo) and Bt exhibit very different acarbose-induced growth lag phenotypes (Fig. 2B; Fig. S3A), we initially hypothesized that a Sus enzyme was differentially inhibited. However, the periplasmic Bo and Bt Sus GH13s and GH97s were inhibited similarly, and the outer membrane α-amylase from Bo, BoSusG, was more inhibited by acarbose than SusG (Table 4). BoSusG and SusG are required for growth on polysaccharides (6, 30, 34). The ability of Bo and Bt to grow on polysaccharides in the presence of acarbose concentrations higher than the BoSusG and SusG IC_50_ values, therefore, suggests that these enzymes are not the primary acarbose target. This suggesting that the drug gets into the periplasm to disrupt maltooligosaccharide processing.

Because deletion of BoSusB and SusB eliminate the acarbose-induced growth lag to the same extent as Δ*sus* deletions when cells are grown on maltose, we propose that these periplasmic enzymes drive the Bo and Bt susceptibility to acarbose albeit to a lesser degree in Bo (Fig. 6B). However, we could not find a singular factor that explains the difference in growth phenotypes between Bo and Bt during acarbose exposure. We suspect that acarbose’s potent upregulation of *sus* might circumvent the lag when the primary target, the periplasmic GH97, is missing (Fig. 8A). Furthermore, we do not know which enzymes support Bo∆BoSusB and Bt∆SusB growth on maltooligosaccharides (Fig. 6A). Although BoGH97D is likely upregulated by larger maltooligosaccharides (Fig. 7), the enzyme may not break down maltoheptaose optimally since Bo∆SusB lags compared to WT Bo when grown on this substrate (Fig. 6A). There are no obvious candidates from the RNAseq data sets comparing ∆Sus and WT transcripts of Bo and Bt grown in maltose to explain how the strains lacking a Sus GH97 grow on maltooligosaccharides. Several SusC/D homologs are upregulated along with some GH18s and GH92s (Tables S4 and 5). GH18 enzymes target chitin (β-linked N-acetylglucosamine) and GH92s are α-mannosidases (49). One or more of these enzymes may be promiscuous and target α-glucans, or α-glucans somehow get modified to become substrates for these enzymes. Despite the pivotal role that the Sus GH97s play in acarbose sensitivity, these enzymes do not explain the vastly different acarbose-induced lag times between Bo and Bt (Fig. 2B).

An additional unexpected point of interaction between acarbose and the *Bacteroides* Sus is the outer membrane transporter. Acarbose likely competes with maltooligosaccharides for passage through the BoSusC/SusC transporter (Fig. 4D). Bo∆SusG and Bt∆SusG exhibit an acarbose-induced growth lag dependent on the size and shape of the oligo they are grown on (Fig. 4). We hypothesize that the lack of growth of cells on alpha-cyclodextrin (αCD) in the presence of acarbose is because acarbose is more readily transported through SusCD. However, this is somewhat surprising given that Bt SusD preferentially binds cyclic oligosaccharides over linear ones (32). SusD-binding affinity may not be equivalent to SusC binding and transport. Additionally, tight binding by SusD may not be advantageous for growth if the “off” rate is too slow to pass the oligo through SusC. This seems unlikely given that both Bo and Bt grow well on αCD (Fig. 4B). Furthermore, acarbose still inhibits growth in Bo∆BoSusC∆BoSusG and Bt∆SusC∆SusG strains, suggesting that the drug can enter the cell independent of BoSusC or SusC (Fig. 4D).

We identified a non-Sus GH97, BoGH97D, that is upregulated by maltose and acarbose in Bo (Fig. 7). Its Bt homolog, BtGH97H, is not upregulated in the same conditions (Fig. 7D). We initially hypothesized that BoGH97D could be responsible for Bo’s relative resistance to acarbose even if the enzyme itself is inhibited by acarbose (Table 3). For instance, BoGH97D could sequester acarbose away from BoSusB. However, Bt does not benefit when grown with acarbose while expressing BoGH97D constitutively (Fig. 7E and F). We do not believe that ectopic expression of BoGH97D was problematic since lysates of Bt∆Sus::BoGH97D have more glucosidase activity than Bt∆Sus (Fig. S13A). SusB is more abundant in the periplasm than BoGH97D, so there may not be enough of the latter to sequester acarbose away from SusB (Fig. 8A). Furthermore, BoSus, as measured by *boSusC* transcript, is upregulated >1,000-fold by maltose plus acarbose compared to glucose alone, while BoGH97D is only upregulated ~15-fold in these conditions (Fig. 7E and 8A). Given that BoGH97D is also inhibited by acarbose, it seems unlikely that its upregulation contributes substantially to the Bo growth phenotype in the presence of acarbose.

Given how potently, compared to maltose, acarbose upregulated *sus* in both organisms, it is possible that maltose is not the strongest *sus* inducer. Indeed, previous work on BtSus showed that maltoheptaose upregulates *sus* at lower concentrations than maltotriose (34). Despite the fact that *bosus* was more upregulated by maltose and acarbose than *btsus*, the SusR regulators do not seem to be the source of the different Bo and Bt acarbose phenotypes since swapping the genes for each protein between the organisms did not swap their acarbose phenotype (Fig. 8B through D). The SusRs are 89% identical, and the SusBs are 93% identical between Bo and Bt. Though it is never safe to assume that proteins with high sequence identity will behave similarly, in the case of the acarbose phenotype described here, we showed this to be a safe assumption.

Though we have not determined a mechanism to explain Bo’s resistance to acarbose, two possibilities, among many, remain. Given that maltose plus acarbose, compared to glucose, upregulated *bosus* fivefold compared to *btsus* and that the SusR proteins do not explain the acarbose phenotype, BoSusC-mediated transport might be faster than SusC-mediated transport. This would lead to faster buildup and increased concentrations of maltooligosaccharides in the Bo periplasm compared to the Bt periplasm. Assessing this possibility will be the goal of future work since it is unknown if BoSusC can pair with SusD and SusC can pair with BoSusD to support growth on starch in Bt and Bo, respectively. This is critical since SusD is required for growth on starch in Bt (32).

Second, though lysates of Bo and Bt grown on maltose with or without 50 µM acarbose break down maltose and maltoheptaose, they do not break down acarbose (Fig. S3B). It is possible that small amounts of acarbose were broken down to products not visible on the TLC, i.e., ones without a reducing end. Further experimentation is necessary to determine if and how either organism can degrade acarbose. However, Bo and Bt cannot use acarbose alone as a carbon source (Fig. S3C).

Lui et al. predicted that acarbose targets intracellular enzymes in their work using fluorescently labeled maltodextrin to determine which bacteria are sensitive to acarbose (25). Species that were sensitive to acarbose-induced growth inhibition still accumulated fluorescent maltodextrin signal in the periplasm in the presence of acarbose. Since this result was also observed in a particular strain lacking a BoSusG or SusG homolog, the authors correctly predicted that acarbose inhibits periplasmic Sus enzymes, which we corroborate here coupled with previous data that SusB is inhibited by acarbose (40, 41). The inhibition of periplasmic enzymes and upregulation of *sus* by acarbose also explains why fluorescent maltodextrin accumulated in the periplasm (25). The authors correctly postulated that the buildup of maltooligosaccharides in the periplasm due to enzyme inhibition would lead to more *sus* upregulation and, therefore, more maltodextrin transport. Our data support this observation, and we would add that acarbose also contributes to this effect because it upregulates *sus* (Fig. 8A) (25). In this case, more *sus* transcript would lead to more BoSusC/SusC transporter at the cell surface, thereby leading to increased maltooligosaccharide import and buildup in the periplasm due to intracellular enzyme inhibition by acarbose (25).

**Fig 9:**
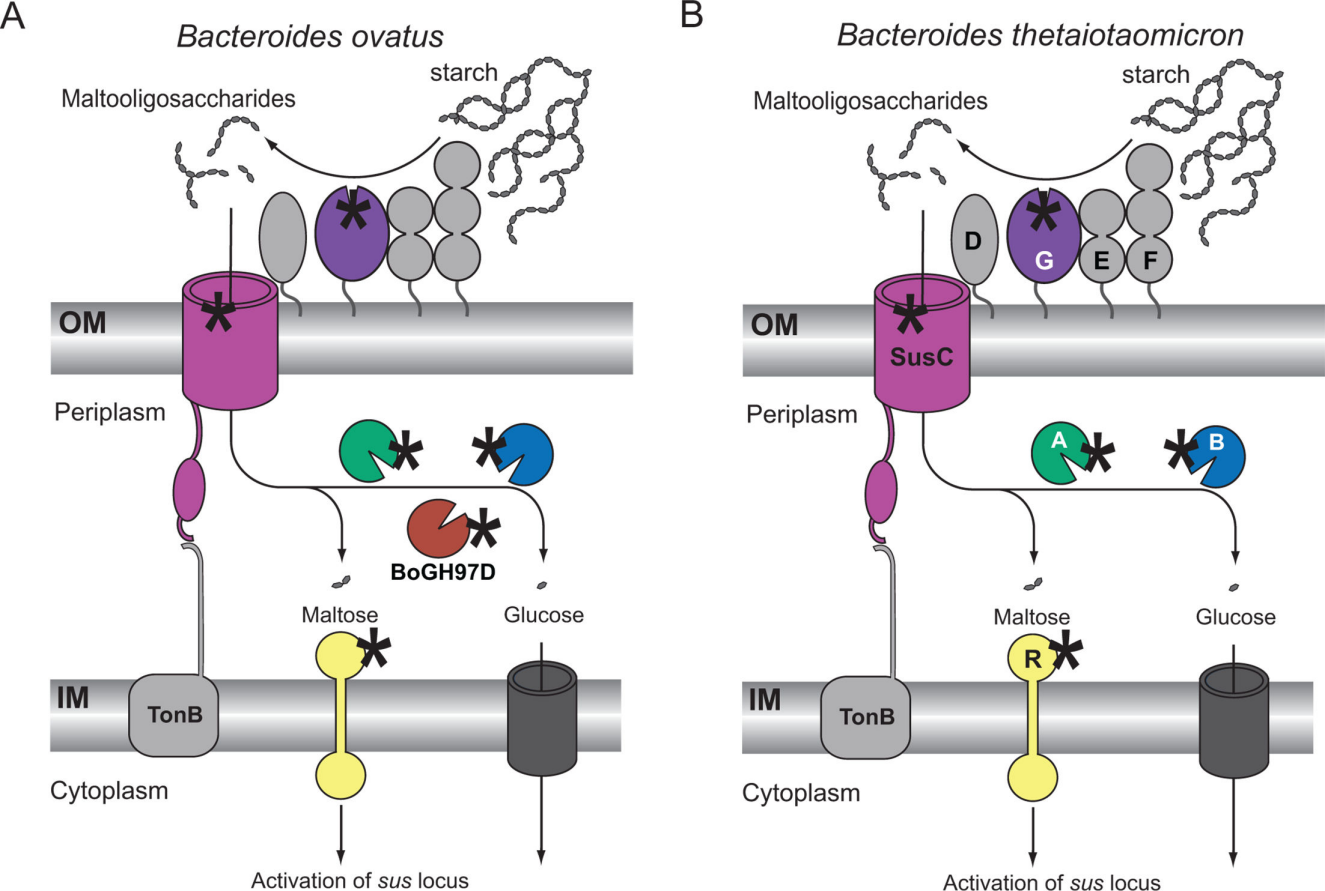
Acarbose targets Bo and Bt Sus components in and outside of the cell. (**A**) Model for how acarbose interacts with BoSus, including the addition of BoGH97D. (**B**) Model for how acarbose interacts with BtSus. Asterisks in panels A and B represent acarbose.

All told, acarbose interacts with Bo and Bt in predicted and unpredicted ways, as modeled in Fig. 9A and B. The results here are akin to what is observed in the *E. coli* K12 mal system. Acarbose is transported by the maltoporin LamB but not appreciably metabolized by the MalQ or MalZ enzymes in *E. coli* (72). *E. coli* does not encode any GH97s in its genome, while MalQ and MalZ are GH77 and GH13 family enzymes, respectively (49). Acarbose, however, is a much more potent inhibitor of *Bacteroides* growth than *E. coli* growth and only weakly upregulates the *mal* system in *E. coli*, unlike the robust *sus* upregulation we observed in Bo and Bt (72).

This newfound mechanistic understanding of how acarbose influences Bacteroidota growth, a phylum implicated in the etiology of diabetes, colorectal cancer, and colitis (73–77), will enable us to predict microbiome responsiveness to acarbose treatment. Furthermore, this work lays a foundation for the design of other xenobiotics with even stronger, or more tailored effects, than acarbose. Given the immense variability of the human gut microbiota, biochemical insight into species-level interactions with acarbose is critical for repurposing this promising drug.

## MATERIALS AND METHODS

### Bacterial genetic manipulation and growth conditions

*Bacteroides ovatus* (ATCC 8483, Bo) and *Bacteroides thetaiotaomicron* (VPI-5482, Bt) were routinely grown in tryptone-yeast extract-glucose (TYG) medium, minimal medium (MM), or brain heart infusion (Becton Dickinson) agar supplemented with 10% horse blood (Colorado Serum Co.) (78, 79). For all growth comparisons and genome manipulations, Bo∆*tdk* and Bt∆*tdk* were used, unless otherwise noted, and are considered wild-type because they contain no *sus* mutations (32, 80). Numerous strains were previously generated via a previously described counter selectable allelic exchange method using a pExchange-*tdk* vector and are listed in Table S6 (30). To “delete” genes encoding the periplasmic Sus enzymes, a premature stop codon was introduced at Leu5 in both GH13s and Ser8 in both GH97s. pExchange plasmids were constructed with 750 bp flanks on either side of the premature stop codon. The gene for BoSusC (*bovatus_03807*) was deleted in both a WT and Bo∆BoGH13A_Sus_ (hereafter referred to as Bo∆BoSusG; Table 1) background. Full BoGH97C_Sus_ (hereafter referred to as BoSusB; Table 1) and SusB deletions were performed in a WT and Bo∆BoSusG and Bt∆SusG background, respectively. These were subsequently used as parent strains to introduce the periplasmic GH97 from the opposite organism to generate Bo∆BoSusB + SusB, Bo∆BoSusB∆BoSusG, Bt∆SusB + BoSusB, and Bt∆SusB∆SusG + BoSusB. The GH97 genes were incorporated in the native *sus* site using 750 bp flanks (*bovatus_03807* and *bovatus_03809*) to introduce SusB into the Bo genome and 750 bp flanks (*susC* and *SusA*) to introduce BoSusB into the Bt genome. The genes for BoSusR (*bovatus_03810*) and SusR (*bt3705*) as well as their promoters (the entire intergenic region between SusR and SusA) were deleted to enable easier construction of the swapped SusR strains. The swapped strains were made by joining the desired SusR and its promoter (up to the SusA start site) to ~750 bp of SusA from the target organism (BoSusR-SusA and SusR-BoSusA) with a single overlap extension (soe-ing) PCR. The insert was cloned into the suicide vector pKnock-*bla-ermG*b (32) and conjugated into Bo or Bt. Colonies were screened for correct orientation of the hybrid SusR-SusA in the *sus* locus.

The gene for BoGH97D (*bovatus_04772*) was cloned into a modified pNBU2 vector with a constitutively active promoter (Bt1311 [sigma 70, *rpoD*]) and complemented in a WT and Bt∆Sus background at the same tRNA^ser^ site to generate Bt::BoGH97D and Bt∆Sus::BoGH97D (70, 79).

All manipulated strains were screened for correct deletion or incorporation of the target gene by PCR-amplifying the expected inserts and sequencing them via Sanger Sequencing using the University of Michigan DNA sequencing core or Azenta Life Sciences. All strains are listed in Table S6, and primers are listed in Table S7.

For plate reader growths for Fig. 1D; Fig. S1, and S2, strains were inoculated in TYG from freezer stocks and grown overnight at 37°C in a Coy anaerobic chamber (85% N_2_/10% H_2_/5% CO_2_). Cells were back diluted 1:50 into enhanced MM containing vitamins, amino acids, purines/pyrimidines, and trace minerals + 5 mg/mL glucose and grown overnight. The following day, cells were pelleted and washed with 2× enhanced MM with no carbon source and diluted 1:100 into enhanced MM + 2.5 mg/mL potato starch and amylopectin with or without 25 µM acarbose in parallel with an enhanced MM + 2.5 mg/mL glucose control. The enhanced MM recipe can be found in reference (81).

For all other growths, strains were inoculated in TYG from freezer stocks and grown overnight at 37°C in a Coy anaerobic chamber (85% N_2_/10% H_2_/5% CO_2_). Cells were back diluted 1:50 into MM + 5 mg/mL glucose, unless otherwise noted, and grown overnight. The following day, cells were pelleted and washed with 2× MM with no carbon source and diluted 1:100 into MM + 2.5 mg/mL carbohydrate with or without acarbose in parallel with a MM + 2.5 mg/mL glucose control. Substrates included maltose (Sigma), maltotriose (Carboexpert), maltoheptaose (Carbosynth), potato amylopectin (Sigma), bovine liver glycogen (Sigma), pullulan (Megazyme), soluble potato starch (Sigma), and α-cyclodextrin (Sigma). Cells were always mixed with a 2× solution of carbohydrate with or without acarbose to achieve the final 1:100 dilution. Kinetic growth experiments were done in 96-well plates in an anaerobic chamber at 37°C on a BioTek Biostack plate-handler and Powerwave HT plate reader. Every 10 min, an OD_600_ of three replicates was recorded.

To calculate acarbose-induced growth lag time, in hours, the average time to an OD_600_ of 0.3 in the −acarbose condition was calculated. This was subtracted from the time to OD_600_ of 0.3 in the +acarbose condition to calculate lag time.

### Gene cloning and site-directed mutagenesis for heterologous protein expression

SusG and BoSusG were previously cloned (6, 36). BoGH13B_Sus_ (hereafter referred to as BoSusA [Table 1] locus tag *bovatus_03809*; NCBI: ALJ48414.1) and SusA (locus tag *bt_3704*; NCBI: AAO78809.1) were cloned using the Lucigen pET-ite system that introduced a TEV cleavage site before a 6× His tag. *Bovatus_03809* encodes a 684 amino acid protein, whereas *bt_3704* encodes a 617 amino acid protein. The first annotation of the *B. ovatus* genome includes a BoGH13B_Sus_ gene (*bacova_03520*) that is 617 amino acids long. Therefore, we assumed this annotation is correct and that the *bovatus_03809* annotation is incorrect. We believe the true start site is at the currently annotated M68 because there is a 22 amino acid sequence following this start that is homologous to the SusA SPI signal peptide (69). SusA was cloned starting with T23 and BoGH13B_Sus_ was cloned starting with A90, hereafter referred to in terms of SusA numbering as A23.

BoSusB (locus tag *bovatus_03808*; NCBI: ALJ48413.1), BoGH97D (locus tag *bovatus_04772*; NCBI: ALJ49360.1), SusB (locus tag *bt_3703*; NCBI: AAO78808.1), and BtGH97H (locus tag *bt_4581*; NCBI: AAO79686.1) were cloned with the Lucigen pET-ite system without a TEV cleavage site since numerous attempts to TEV cleave these proteins were unsuccessful and previous work with SusB was done with enzyme retaining a 6× N-terminal His tag (40, 41). All GH97s here were cloned with an N-terminal 6× His tag.

BoSusA and SusA genes were mutated to introduce a D to N mutation at position 331. This position aligns with the catalytic nucleophile in other GH13 enzymes. Site-directed mutagenesis was performed via splicing by overlap-extension PCR (SOE-ing PCR).

All constructs were confirmed by Sanger Sequencing at the University of Michigan Sequencing Core or Azenta Life Sciences. Primers used during construct cloning are listed in Table S7.

### Heterologous protein expression and purification

Rosetta (DE3) pLysS cells were transformed with the appropriate construct and grown overnight in LB. 10 mL of LB per 500 mL of TB was used to inoculate TB medium the following morning (0.5 L for all GH97s and 1 L for all GH13s). Cells were grown to an OD_600_ of 0.6–0.7 at 37°C and moved to room temperature to shake for 0.5 h before protein expression was induced with the addition of 0.5 mM isopropyl β-d-1-thiogalactopyranoside (IPTG). Cells were grown at room temperature (~22°C) for 18 h and harvested by centrifugation for 10 min at 10,000 *× g*. Cell pellets were stored at −80°C. All proteins were purified according to reference 6 except that the GH97s were dialyzed against storage buffer (20 mM HEPES, 100 mM NaCl, pH 7.0) after the first purification because they were not TEV-cleaved.

### BoSusA and SusA size exclusion chromatography

One-half milliliter of 10.6 mg/mL BoSusA or 8.2 mg/mL SusA was applied to a HiPrep 16/60 Sephacryl S-200 HR (GE Healthcare) in 20 mM HEPES, 100 mM NaCl, pH 7.0, at a flow rate of 0.5 mL/min. BioRad gel filtration standards were reconstituted in 0.5 mL of the same buffer and run using the same protocol for comparison (bovine thyroglobulin [670 kDa], bovine γ-globulin [158 kDa], chicken ovalbumin [44 kDa], horse myoglobin [17 kDa], vitamin B12 [1.35 kDa]).

### α-Glucan breakdown by cell lysates

Bo, Bo∆Sus, Bt, Bt∆Sus, Bt::BoGH97D, and Bt∆Sus::BoGH97D were grown anaerobically in TYG from freezer stocks and back diluted into MM + 5 mg/mL maltose. Strains were back diluted to an OD_600_ of 0.2 in 10 mL of MM + 5 mg/mL maltose without acarbose and with 50 µM acarbose (for Bo and Bt only). Bacteria were grown to an OD_600_ of 0.7 and pelleted via centrifugation for 10 m at 5,000 rpm. Supernatant was decanted, and cells were washed 2× in PBS. Cell pellets were flash frozen in liquid nitrogen and stored at −80°C. Pellets were lysed in 1 mL of PBS via sonication. Half of the lysate was retained, and half was pelleted for 10 m at 13,000 × *g*. The pellet was discarded, and the clarified lysate was retained. Clarified and unclarified lysates were mixed 1:1 with 10 mg/mL maltose, maltoheptaose, or acarbose and incubated overnight at 37°C. Reaction products were analyzed via thin-layer chromatography, described below.

### Thin-layer chromatography

To assess oligosaccharide and polysaccharide break down by enzymes, thin-layer chromatography (TLC) was performed as described in reference 6. 0.5 µM protein and 5 mg/mL of one of the following carbohydrates were used: maltose (Sigma), maltotriose (Carboexpert-BoSusA/SusA, Sigma-GH97s), maltotetraose (Carboexpert), maltopentaose (Carbosynth), maltohexaose (Carbosynth), maltoheptaose (Carboexpert), 6^3^-α-d-glucosyl-maltotriose (Megazyme), 6^3^-α-d-glucosyl-maltotriosyl-maltotriose (Megazyme), α-cyclodextrin (Sigma), β-cyclodextrin (Sigma), acarbose (Sigma), acarviosin (CarboSynth) d-panose (Sigma), isomaltose (Sigma), potato amylopectin (Sigma), bovine liver glycogen (Sigma), pullulan (Sigma), soluble potato starch (Sigma), and dextran (Sigma). All polysaccharides were autoclaved. A 20 mM HEPES, 100 mM NaCl (pH 7.0) buffer was used for BoSusA and SusA, while 100 mM maleate, 10 mM CaCl_2_ (pH 6.6) was used for BoSusB, BoGH97D, SusB, and BtGH97H according to conditions defined in reference 41. Two microliters of each reaction, as well as a no enzyme control, was spotted for separation and a 1 mg/mL solution of G1–G7 in the appropriate reaction buffer was used as a ladder.

To resolve lysate reactions with maltose, maltoheptaose, or acarbose, 2 µL of each reaction, as well as a no lysate control, was spotted for separation and a 1 mg/mL solution of G1–G7 in PBS was used as a ladder, along with acarbose and acarviosin in PBS as a comparison. TLC plate running and visualization conditions are described in reference 6.

### Enzyme kinetics

BoSusG and SusG inhibition was quantified using the EnzChek *Ultra* Amylase Assay Kit (Thermo). Briefly, the starch substrate in this assay is modified with a BODPY FL dye such that fluorescence is quenched. Upon digestion, the quenching is relieved and yields fluorescent fragments. The kit was used according to the manufacturer’s instructions except that a final concentration of 0.2 mg/mL fluorescent starch was used. 2× BoSusG was preincubated with or without 2 × 0.5–10 µM acarbose for 10 min, and 2× SusG was preincubated with or without 2 × 5–1,000 µM acarbose for 10 min. Reactions were initiated via the mixing of 50 µL of 2× protein/buffer/acarbose and 50 µL of 2× starch for final concentrations of 25 nM enzyme, 0.2 mg/mL starch, and the previously indicated acarbose concentrations in 20 mM HEPES, 100 mM NaCl, pH 7.0. Reactions were performed in duplicate in black 96-well plates with clear bottoms (Corning) and monitored every 70 s via excitation at 485 nm and emission at 528 nm in a Synergy H1 Plate Reader. Initial rates were calculated between 6 and 15 min. The 0 µM acarbose condition was treated as 100% activity, and percent activity as a function of [acarbose] was graphed and analyzed in GraphPad Prism with a non-linear [inhibitor] vs normalized response model to calculate an IC_50_.

BoSusA and SusA Michaelis-Menten and inhibition kinetics were quantified using pNP-G6 (Sigma) in 20 mM HEPES, 100 mM NaCl, pH 7.0. Initial Michaelis-Menten parameters were determined with 1 nM enzyme in 25–750 µM pNP-G6 in duplicate. pNP release was monitored every minute at 405 nm in a Synergy H1 plate reader. Initial rates were calculated between 0 and 10 min. The *K*_*M*_ was subsequently used to inform inhibition conditions. Enzymes were pre-mixed with acarbose for 10 min and mixed 1:1 with pNP-G6 in duplicate. 60, 125, 250, 375, and 625 µM pNP-G6 was used with 0–480 nM acarbose and 2 nM enzyme. Inhibition was initially modeled in GraphPad Prism using a mixed model of inhibition. The subsequent low, but not zero, α value led us to do a final model using uncompetitive inhibition with an α*K*_*i*_ reported.

All GH97 (BoSusB, BoGH97D, SusB, and BtGH97H) kinetics were quantified using pNP-α-Glc (pNP-Glc, Sigma) in 100 mM maleate, 10 mM CaCl_2_, pH 6.6 according to reference 41. Initial Michaelis-Menten parameters for BoSusB and SusB were used with 10 nM enzyme, while 2 nM BoGH97D and BtGH97H was used with 10–500 µM pNP-Glc in duplicate. pNP release was monitored as described above, and initial rates were determined between 0 and 10 min. The *K*_*M*_ was subsequently used to inform inhibition conditions. Enzymes were pre-mixed with acarbose for 10 min and mixed 1:1 with pNP-Glc in duplicate. 50, 100, 200, 300, and 500 µM pNP-Glc and 0–1,000 µM acarbose were used. pNP release was monitored as described above, and initial rates were determined between 0 and 10 min. GraphPad Prism was used to model competitive inhibition in all four enzymes to derive a *K*_*i*_.

### Structure of BoSusB bound to acarbose

BoSusB (9 mg/mL) (using an estimated extinction coefficient of 156,268 M^−1^cm^−1^) with 10 mM acarbose was initially screened via sitting drop vapor diffusion at room temperature with an Art Robbins Gryphon LCP robot. Commercially available kits from Molecular Dimensions and Hampton were used for screening. Hanging drop refinement was carried out with a Molecular Dimensions Morpheus screen condition that included 100 mM NaHEPES/MOPS (pH 7.5; Thermo), 100 mM amino acids (from commercially available stock—includes l-Na-glutamate, racemic alanine, glycine, racemic lysine-HCl, and racemic serine), and 36% precipitant mix 4 (from commercially available stock 1:1:1 mix of racemic MPD, polyethylene glycol 1K, and polyethylene glycol 3350). Drops were a 1:1 mix of protein/acarbose:well solution. Suitable crystals were cryo-protected in a mix comprised of 70% well solution, 20% ethylene glycol, and 10% acarbose (final concentration of 10 mM) for 30 s before flash-freezing in liquid nitrogen. These crystals belonged to the orthorhombic space group *P*2_1_2_1_2_1_ with unit cell dimensions of *a* = 107.79 Å, *b* = 116.51 Å, *c* = 143.96 Å, α,β,γ = 90°. X-ray diffraction data were collected at the Advanced Photon Source Life Science Collaborative Access Team (LS-CAT) beamline 21 ID-G at Argonne National Laboratories. Data were processed and scaled in Xia2 with DIALS followed by molecular replacement in Phenix with PDB ID: 2ZQ0 (SusB) as a model (40, 82–84). Autobuild produced an initial structure with two monomers in the asymmetric unit (85). The model was manually adjusted in Coot followed by refinement in Refmac (86, 87). Acarbose geometry was validated using Privateer (88).

### Isothermal titration calorimetry

Acarbose binding to BoSusA and SusA was assessed via isothermal titration calorimetry using a TA instruments standard volume ITC. All experiments were performed in triplicate at 25°C using 25 µM BoSusA, BoSusA-D331, SusA, and SusA-D331N with 3.5 mM acarbose for the Bo proteins and 3 mM acarbose for the Bt proteins. A constant blank correction was used to account for the heat of dilution, and all data were analyzed with an independent binding model using the manufacturer’s NanoAnalyze software.

### pNP-Glc activity by cell lysates

Bo, Bo∆Sus, Bt, Bt∆Sus, Bt::BoGH97D, and Bt∆Sus::BoGH97D were grown in TYG overnight from freezers stocks and back diluted into MM + 5 mg/mL maltose. The next morning, they were back diluted to an OD_600_ of 0.2 in 10 mL MM + 5 mg/mL maltose and grown to an OD_600_ of 0.7. Cells were pelleted, washed 2× with PBS, pelleted, and flash frozen in liquid nitrogen and stored at −80°C. Pellets were lysed in 1 mL of PBS via sonication. Debris was pelleted for 10 m at 13,000 *× g,* and the clarified lysate was retained. Glucosidase activity was monitored with a 1:4 dilution of lysate in 1 mM pNP-Glc with or without 1 µM acarbose in PBS in duplicate. pNP release was monitored every minute at 405 nm in a Synergy H1 plate reader. Rates were calculated between 0 and 10 min.

### Activity guided fractionation

Bo∆Sus was first inoculated into TYG from a freezer stock and then back diluted, the next day, 1:100 in 50 mL of MM + 5 mg/mL maltose. The following morning, all 50 mL were used to inoculate 1 L of MM + 5 mg/mL maltose. Cells were grown to an OD_600_ of 0.75 and subsequently pelleted for 10 m at 22,000 × *g* at 4°C. The pellet was stored at −80°C for further processing. The Bo∆Sus pellet was resuspended in 80 mL of PBS, and cells were lysed via sonication. The lysate was clarified via centrifugation at 30,000 × *g* for 30 m at 4°C. 7.69 g ammonium sulfate was added to 70 mL of clarified lysate to achieve 20% saturation and spun for 1 h at 4°C. The mixture was centrifuged for 15 m at 10,000 × *g* at 4°C. Twenty milliliters of the supernatant was saved for dialysis overnight against PBS. The process was repeated with the appropriate amount of solid ammonium sulfate to obtain 20 mL of mixtures at 30% and 50% and 10 mL at 70% saturation. All samples were dialyzed against 4 L of PBS at 4°C overnight.

Fifty microliters of each sample was mixed with 50 µL of 10 mM pNP-Glc to check for α-glucosidase activity. Fifty percent ammonium sulfate saturation still afforded activity while 70% did not, so the protein sample from 50% saturation was used for downstream processing. The protein sample, in PBS, was applied to a 5 mL HiTrap Q FF column (Cytiva) preequilibrated in PBS. The column was washed and a 70 mL gradient from 100% 1× PBS (containing 137 mM NaCl and 2.7 mM KCl) to 100% PBS with extra salt in the same ratio (980 mM NaCl, 19.6 mM KCl). Only flow through fractions contained pNP-Glc activity, so they were pooled, concentrated, and applied to a HiPrep 16/60 Sephacryl S-200 HR (GE) preequilibrated in PBS. Flow-through fractions were assessed for pNP-Glc activity and those with activity were pooled. They were applied to a 5 mL HiTrap SP FF column (Cytiva) in PBS and after dialyzing against 2-(N-morpholino)ethanesulfonic acid buffer at pH 6. No protein stuck to the column in either buffer. Active fractions were pooled and dialyzed against NaPO_4_ at either pH 5.1 or pH 8.4 to apply to a Q column equilibrated in the appropriate buffer. At pH 8.4, protein with pNP-Glc activity stuck to the column and was eluted in a gradient from 0 to 1 M NaCl in NaPO_4_. Active fractions were concentrated to 100 µL after dialysis overnight in 20 mM Bis-Tris propane (pH 8), 50 mM NaCl.

Samples were submitted to Proteomics Resource Facility at University of Michigan for analysis. Briefly, cysteines were reduced with 10 mM DTT (45°C for 30 min) and alkylated with 65 mM 2-chloroacetamide, without light, for 30 min at room temperature. An overnight digestion with 1 µg sequencing grade modified trypsin was carried out at 37°C with constant mixing (ThermoMixer). The digestion was stopped by acidification, and the peptides were desalted using SepPak C18 cartridges using the manufacturer’s protocol (Waters). Samples were then completely dried using a vacufuge. The resulting peptides were dissolved in 9 µL of 0.1% formic acid/2% acetonitrile solution. Two microliters of the resulting peptide solution was resolved on a nano-capillary reverse phase column (EasySpray PepMap C18, 2 µm, 50 cm, #ES903, ThermoScientific) using a 0.1% formic acid/acetonitrile gradient at 300 nL/min over a period of 180 min. The eluent was directly introduced into a Q Exactive HF mass spectrometer (Thermo Scientific, San Jose CA) using an EasySpray source. MS1 scans were acquired at 60K resolution (AGC target = 3 × 106; max IT = 50 ms). Data-dependent collision-induced dissociation MS/MS spectra were acquired on 20 most abundant ions following each MS1 scan (NCE ~28%; AGC target 1 × 105; max IT 45 ms).

Proteins were identified by searching the data against the *Bacteroides ovatus* protein database (5,499 entries) using Proteome Discoverer (v2.1, Thermo Scientific). Search parameters included MS1 mass tolerance of 10 ppm and fragment tolerance of 0.05 Da; two missed cleavages were allowed; carbamidomethylation of cysteine was considered fixed modification and oxidation of methionine, deamidation of asparagine and glutamine, variable modifications. False discovery rate (FDR) was determined using Percolator, and proteins/peptides with an FDR of ≤1% were retained for further analysis.

### GH97 sequence analysis

Bo and Bt GH97 amino acid sequences according to the latest version of CAZy.org (49) were aligned in MegAlignPro using MAFFT (89, 90). A phylogenetic tree was made in MegAlignPro using the Neighbor-Joining algorithm and visualized in iTOL (91, 92). BoSusB, BoGH97D, SusB, and BtGH97H amino acid sequences were aligned and visualized using ClustalOmega on the EMBL-EBI server (93, 94).

### RNAseq analysis

Bt, Bo, Bt∆Sus, and Bo∆Sus were grown anaerobically in TYG from freezer stocks and back diluted into MM + 5 mg/mL maltose. Cells, in triplicate, were back diluted to an OD_600_ of 0.1 in 5 mL MM + 5 mg/mL maltose and grown to an OD_600_ of 0.5. 10 mL of RNAprotect Bacteria Reagent (Qiagen) was added to cultures, incubated for 1 m, and cells were pelleted for 10 m at 3,500 rpm. Supernatant was removed, and the pellets were stored at −80°C until further processing.

Total RNA was purified according to reference 95 followed by treatment with DNase I (Invitrogen). RNA was repurified via standard sodium acetate/isopropanol precipitation and quantified using a NanoDrop (ThermoFisher). RNA, in water, was frozen at −80°C. rRNA depletion, library preparation, and sequencing were subsequently performed by SeqCenter (Philadelphia).

Raw reads were first trimmed and filtered via TrimGalore (version 0.6.6) and then aligned to the Bo or Bt protein-coding sequences via the DIAMOND aligner (version 2.0.6). Reads aligning to multiple protein-coding references were adjudicated via FAMLI https://github.com/Golob-Minot/FAMLI2 [96]). The resultant specimen-gene-count matricies were outputted in anndata format. The entire workflow from raw reads to anndata count matricies was implemented in Nextflow and available at https://github.com/jgolob/transcriptshot.

Differential gene expression was determined via Student *t*-tests, with Benjamini-Hochberg correction for false discovery. We used the following parameters to delineate significant up or down regulation: one mean ≥ 10 rpm; false discovery corrected students *t*-test *P* value ≥ 0.05; fold change ≥ 2.

### qPCR

Bo and Bt were grown in triplicate as described for the RNAseq analysis in MM + 5 mg/mL glucose or maltose with or without 50 µM acarbose. Total RNA was extracted from cells using an RNeasy kit (Qiagen), treated with DNase I (NEB), and reverse transcribed using Superscript III (Invitrogen) according to manufacturer’s protocols. The 16S rRNA gene-normalized transcript abundances were assayed on a Bio-Rad CFX Connect thermocycler using a custom qPCR master mix (97).

## Data Availability

RNAseq data were deposited under the NIH Sequence Read Archive BioProject PRJNA1107240. Proteomics data were deposited in the ProteomeXchange Consortium via the Proteomics IDEntifications Database (PRIDE) partner repository with the data set identifier of PXD052070 (98). The BoSusB–acarbose structure was deposited with the RCSB Protein Databank with a PDB ID of 9BS5. All plasmids, proteins, bacterial strains, and other reagents generated for this work will be made freely available to researchers using them for non-commercial reasons.

## SUPPLEMENTAL MATERIAL

Supplemental Figures mbio.01506-24-s0001.docx Figures S1-S14.

Legends mbio.01506-24-s0002.docx Legends for supplemental tables.

Table S1 mbio.01506-24-s0003.docx BoSusA and SusA ITC with acarbose.

Table S2 mbio.01506-24-s0004.docx BoSusB X-ray data and refinement statistics.

Table S3 mbio.01506-24-s0005.xlsx Proteomics data.

Table S4 mbio.01506-24-s0006.xlsx Bo RNAseq data.

Table S5 mbio.01506-24-s0007.xlsx Bt RNAseq data.

Supplemental tables mbio.01506-24-s0008.docx Tables S6 and S7.

ASM does not own the copyrights to Supplemental Material that may be linked to, or accessed through, an article. The authors have granted ASM a non-exclusive, world-wide license to publish the Supplemental Material files. Please contact the corresponding author directly for reuse.
