# Supplementary material for: Acarbose impairs gut Bacteroides growth by targeting intracellular glucosidases
Source: mBio. 2024 Nov 20;15(12):e01506-24. doi: 10.1128/mbio.01506-24 (PMC11633381; doi:10.1128/mbio.01506-24)
Supplement: Supplemental Figures — Figures S1-S14. [file mbio.01506-24-s0001.docx]

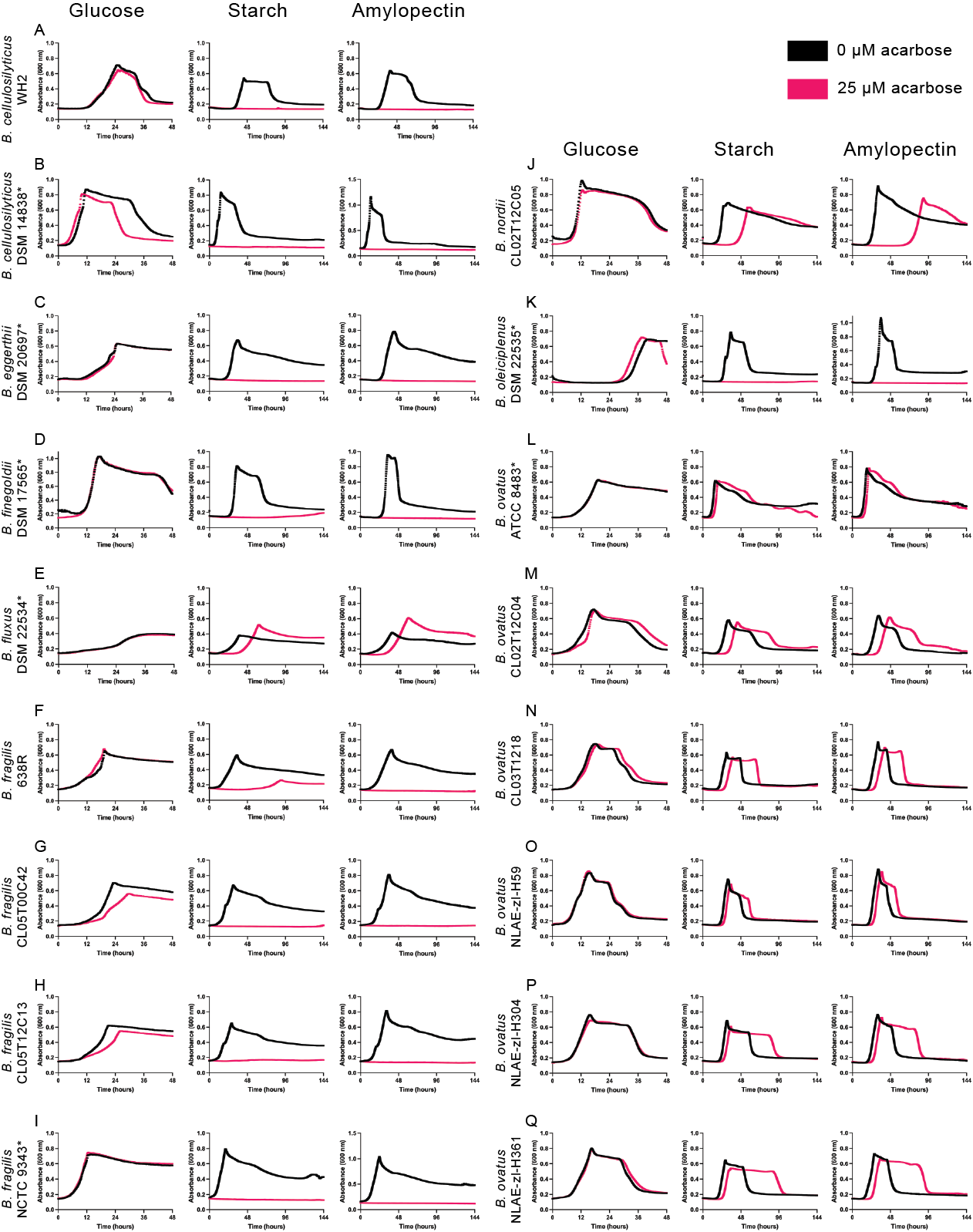


**Supplementary Figure 1. Bacteroidota species exhibit different susceptibilities to acarbose induced growth inhibition.** The indicated species were pre-grown in enhanced minimal media (MM) with glucose and back diluted into enhanced MM + 2.5 mg/ml of the indicated carbon sources with and without 25 µM acarbose. Growths were performed in triplicate and the average is displayed.


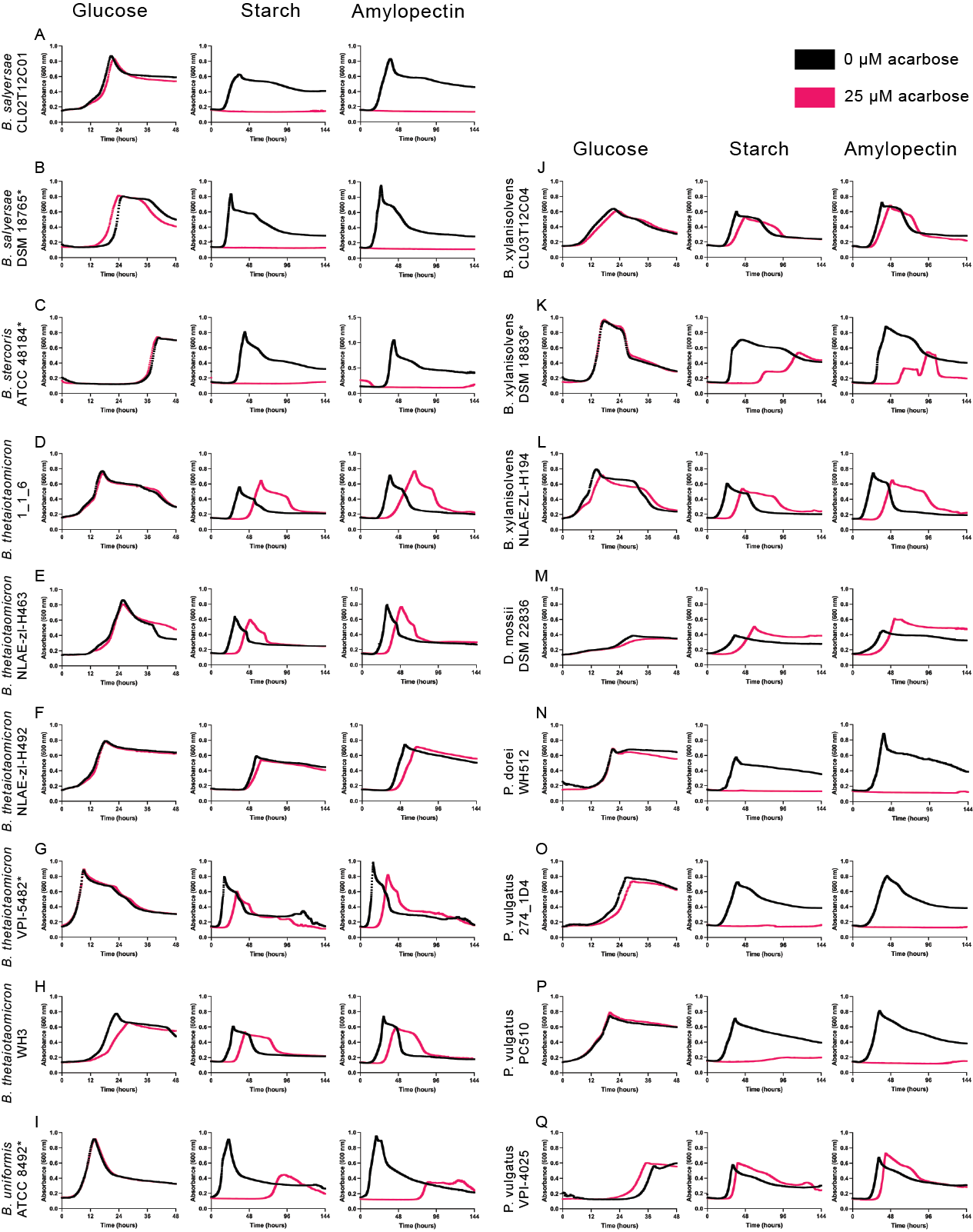


**Supplementary Figure 2. Bacteroidota species exhibit different susceptibilities to acarbose induced growth inhibition.** The indicated species were pre-grown in enhanced minimal media (MM) with glucose and back diluted into enhanced MM + 2.5 mg/ml of the indicated carbon sources with and without 25 µM acarbose. Growths were performed in triplicate and the average is displayed.


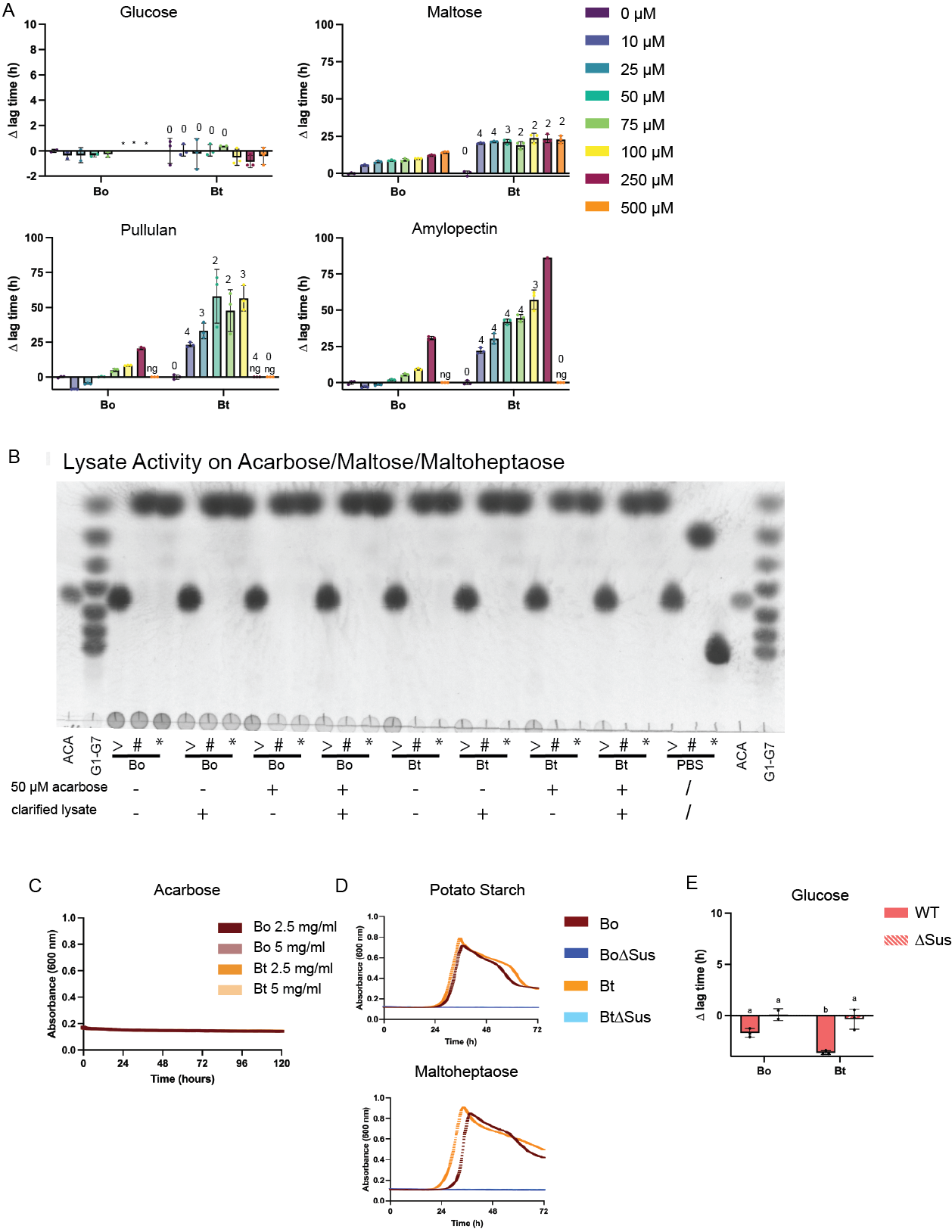


**Supplementary Figure 3. Bt is more susceptible to acarbose induced growth inhibition than Bo.** A) Bo and Bt were pre-grown in minimal media (MM) with glucose and back diluted into MM + 2.5 mg/ml of the indicated carbon sources with and without 10 – 500 µM acarbose. The difference in time to OD_600_ of 0.3 between the treated and untreated conditions are graphed. Bo and Bt with the same treatment were compared using an unpaired, two-tailed Student’s *t* test. 1: p ≤ 0.05; 2: p ≤ 0.01; 3: p ≤ 0.001. 4: p ≤ 0.0001. ng = no growth. Technical difficulties with the plate reader did not allow us to calculate an acarbose induced lag time at an OD_600_ of 0.3 for conditions marked with *. Growths were performed in triplicate. The mean and standard deviation are displayed for conditions in which at least two replicates grew. Statistics are not included for conditions in which only one replicate grew. B) Bo and Bt were grown in MM + 5 mg/ml maltose with or without 50 µM acarbose to an OD_600_ of 0.7. Cells were pelleted and washed with PBS and sonicated to release cellular contents. Lysates and clarified lysates were incubated overnight at 37 °C with 5 mg/ml acarbose (>), maltose (#), or maltoheptaose(*).Clarified lysates were pelleted following cell sonication to remove insoluble components. No lysate controls (PBS alone) were also performed. 1 mg/ml acarbose and 1 mg/ml G1-G7 in PBS were run out on a thin layer chromatography (TLC) plate as reference points. C) Acarbose growth curves D) WT and ∆Sus strains of Bo and Bt were pre-grown as described in A and back diluted into MM + 2.5 mg/ml amylopectin or maltoheptaose. E) WT and ∆Sus strains of Bo and Bt were pre-grown as described in A and back diluted into MM + 2.5 mg/ml glucose with or without 50 µM acarbose. The difference in time to OD_600_ of 0.3 between the treated and untreated conditions (∆ lag time) is graphed. Statistical analyses were performed using a two-way ANOVA with a cutoff of p≤0.05. Condition(s) with the same letter were not statistically different from one another. Growths were performed in triplicate. The mean and standard deviation are displayed.


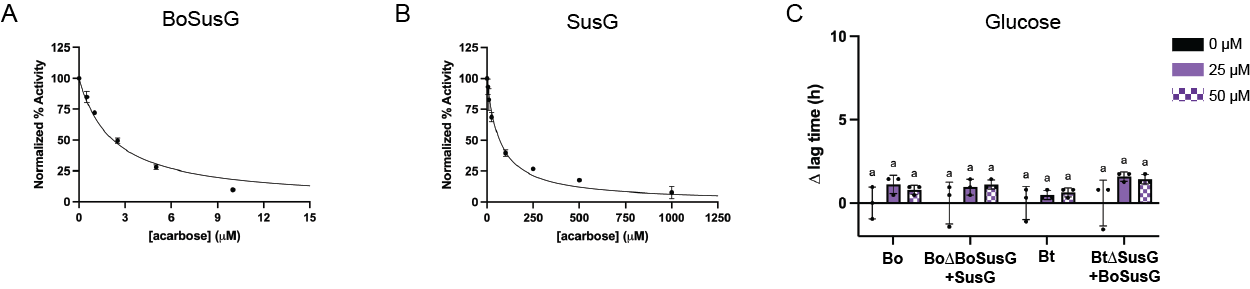


**Supplementary Figure 4. Outer membrane amylases BoSusG and SusG are not the source of the acarbose lag phenotype.** A,B) BoSusG and SusG starch breakdown in the presence of various acarbose concentrations was assessed using an EnzChek™ *Ultra* Amylase Assay Kit from Thermo. 25 nM enzyme and 0.2 mg/ml fluorescent starch was used. Activity in the absence of acarbose was set to 100% and percent activity of this was graphed vs. acarbose concentration to calculate IC_50_ values, reported in Table 4. C) Because BoSusG does not optimally complement Bt∆SusG growth on starch, these growths were performed with bacteria pre-grown on minimal media (MM) + 5 mg/ml maltose to induce *sus* expression, then inoculated into MM + 2.5 mg/ml glucose in the indicated acarbose concentrations as controls. The difference in time to OD_600_ of 0.3 between the treated and untreated conditions (∆ lag time) is graphed. Statistical analyses were performed using a two-way ANOVA using a cutoff of p≤0.05. Conditions with the same letter(s) were not significantly different from one another. All growths were performed in triplicate. The mean and standard deviation are shown.


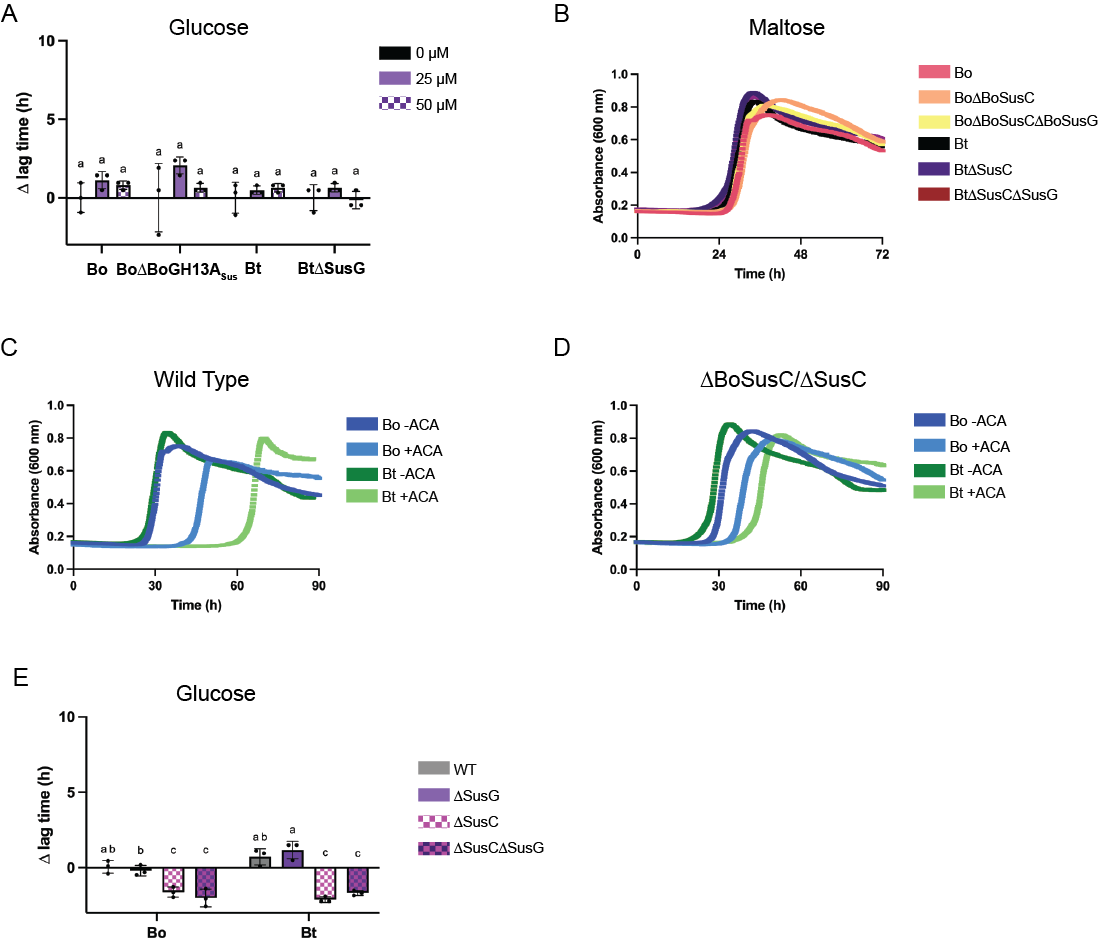


**Supplementary Figure 5. Acarbose likely competes with maltooligosaccharides for transport through BoSusC and SusC.** A) Bacteria were pre-grown in minimal media (MM) with glucose and back diluted in MM + 2.5 mg/ml glucose with and without the indicated acarbose concentrations. The difference in time to OD_600_ of 0.3 between the treated and untreated conditions (∆ lag time) is graphed. B) Bacteria were pre-grown in MM with glucose and back diluted in MM + maltose. C,D) Bacteria were pre-grown in MM with glucose and back diluted into MM + maltose with or without 50 µM acarbose (ACA). E) Bacteria were pre-grown in minimal media (MM) with glucose and back diluted in MM + 2.5 mg/ml glucose with and without 50 µM acarbose. The difference in time to OD_600_ of 0.3 between the treated and untreated conditions (∆ lag time) is graphed. Statistical analyses in A and C were performed with a two-way ANOVA. Conditions with the same letter(s) were not significantly different from one another. A cutoff of p≤0.05 was used. All growths were performed in triplicate. The mean and standard deviation are shown in the ∆ lag graphs while the growth curves show the average of all three growths.


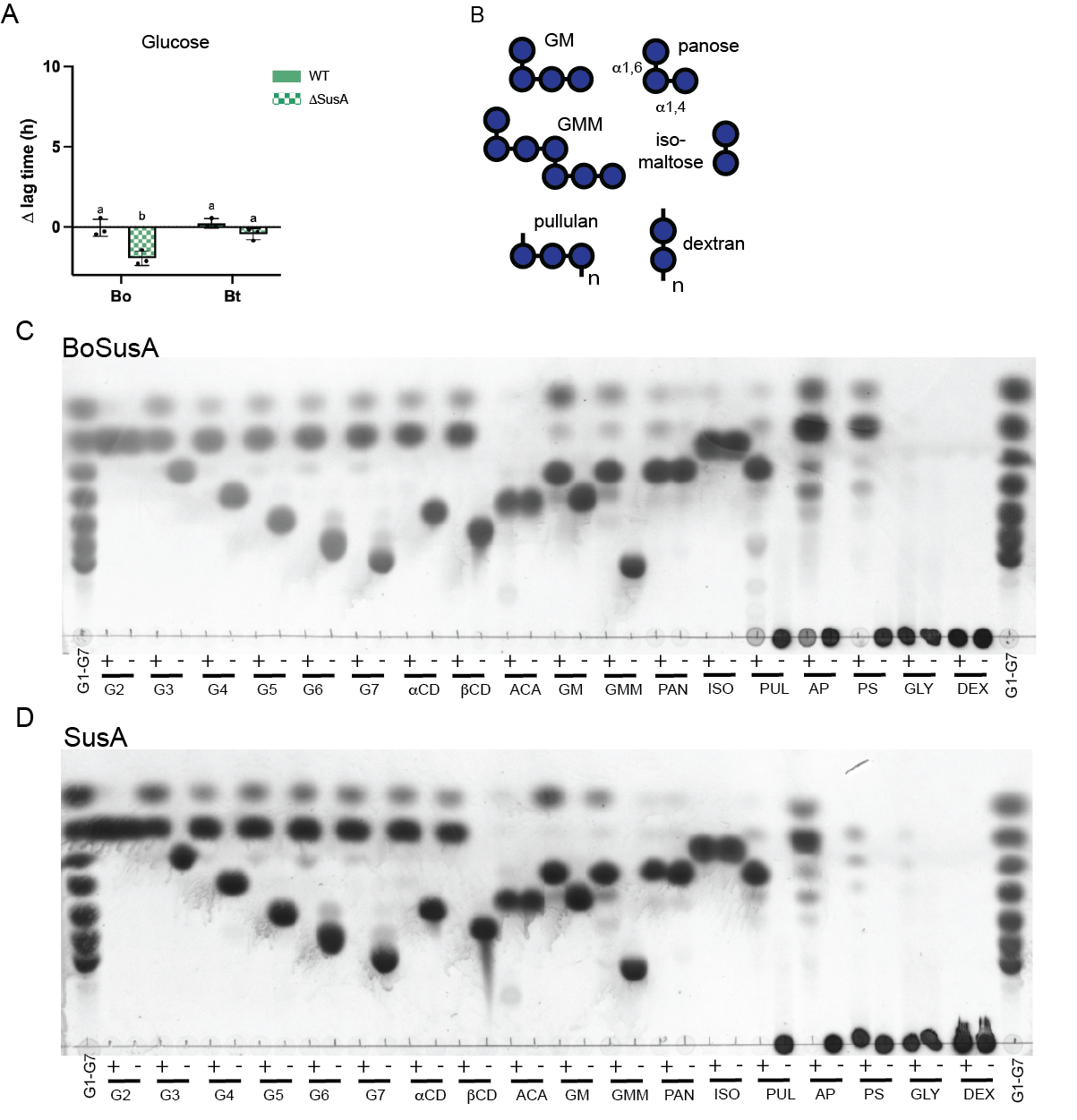


**Supplementary Figure 6. BoSusA and SusA do not underpin the different Bo and Bt acarbose phenotypes and have nearly identical substrate preferences.** A) Bo and Bt were pre-grown in minimal media (MM) with glucose and back diluted into MM + 2.5 mg/ml glucose with or without 50 µM acarbose. The difference in time to OD_600_ of 0.3 between the treated and untreated conditions (∆ lag time) is graphed. Statistical analyses were performed with a two-way ANOVA. Conditions with the same letter(s) were not significantly different from one another. A cutoff of p≤0.05 was used. All growths were performed in triplicate. The mean and standard deviation are shown. B-C) 500 nM of the indicated enzyme was incubated overnight with 5 mg/ml of the following carbohydrates: G2 – maltose; G3 – maltotriose; G4 – maltotetraose; G5 – maltopentaose; G6 – maltohexaose; G7 – maltoheptaose; αCD – alpha-cyclodextrin; βCD – beta-cyclodextrin; ACA – acarbose; GM – 6^3^-α-d-glucosyl-maltotriose; GMM – 6^3^-α-d-glucosyl-maltotriosyl-maltotriose; PAN – d-panose; ISO – isomaltose; PUL – pullulan; AP – potato amylopectin; PS – potato starch; GLY – glycogen; DEX – dextran. All polysaccharides were autoclaved to get them into solution. + = with enzyme. - = no enzyme control.


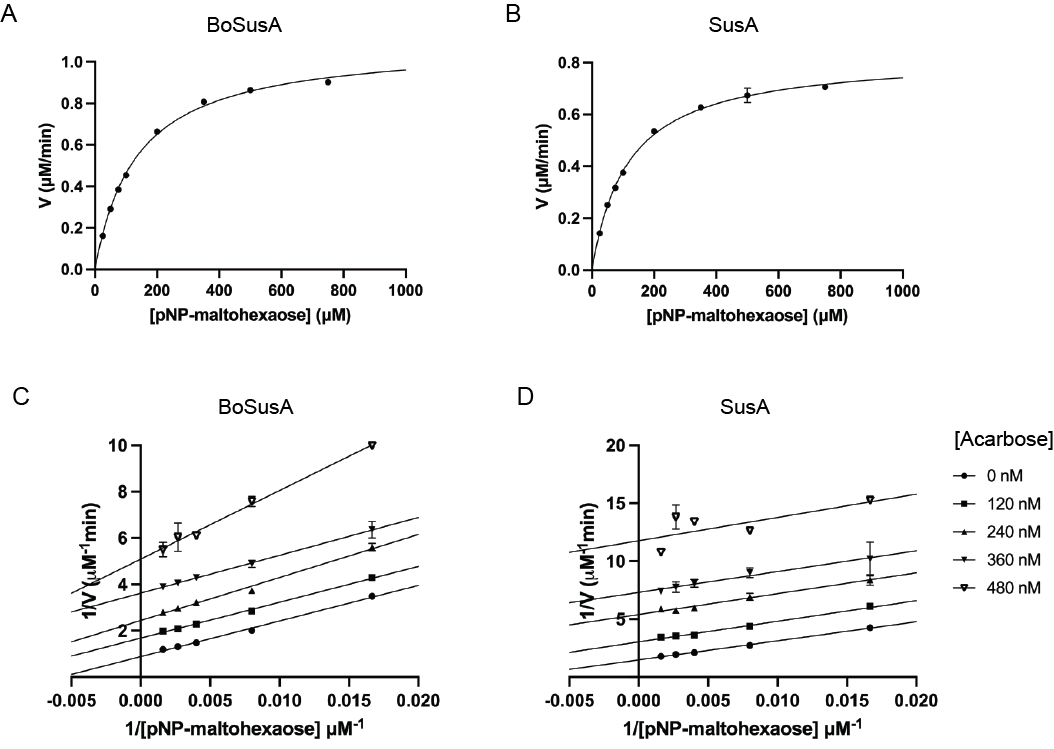


**Supplementary Figure 7. BoSusA and SusA have similar catalytic efficiencies and are inhibited similarly by acarbose.** A,B) Enzymes were incubated for 10 min in various concentrations of pNP-maltohexaose in duplicate. Initial rate as a function of substrate concentration is shown. C,D) Enzymes were pre-incubated for 10 min with acarbose before mixing with various pNP-maltohexaose concentrations. Initial rates were recorded and used to make the double reciprocal Lineweaver-Burk plots shown. Michaelis-Menten and inhibition parameters can be found in Table 4. 1 nM of each enzyme was used for both experiments.


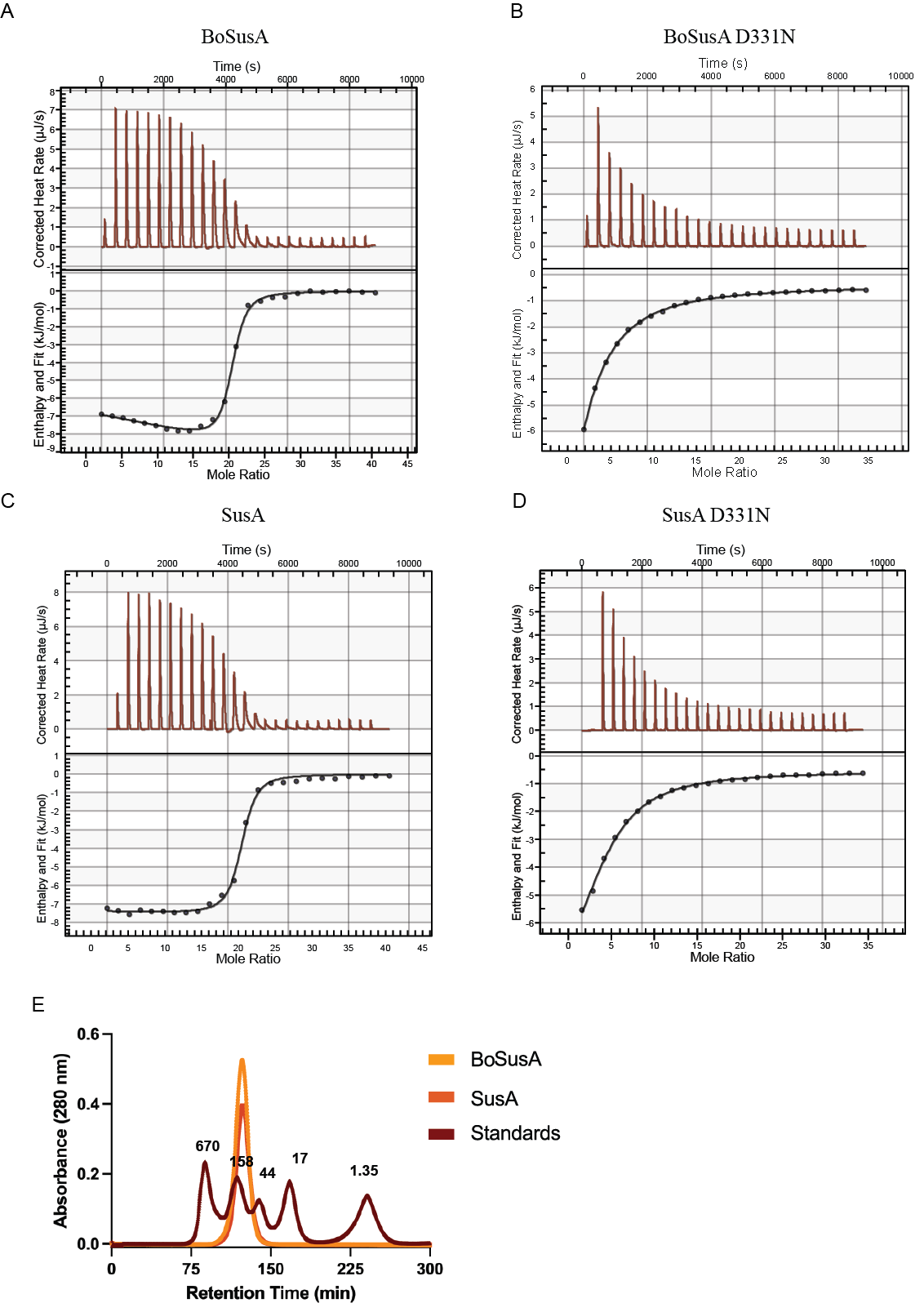


**Supplementary Figure 8. BoSusA and SusA are both dimers that bind tightly to acarbose in the absence of substrate.** A-D) 25 µM of each enzyme was assessed for binding to 3.5 mM (Bo enzymes) or 3 mM (Bt enzymes) using a standard volume isothermal titration calorimeter from TA Instruments. Experiments were performed in triplicate with the average *K*_D_ and n values being reported in Supplementary Table 1. Representative curves are displayed for each condition. E) BoSusA and SusA were applied to a HiPrep 16/60 Sephacryl S-200 HR column. Monomers of each enzyme are expected at ~69 kDa and dimers at ~138 kDa. Comparing the retention times of each enzyme to a standard curve of the logarithm of the retention time of the standards led to an estimated molecular weight of 125 kDa for BoSusA and 121 kDa for SusA, consistent with dimer formation.


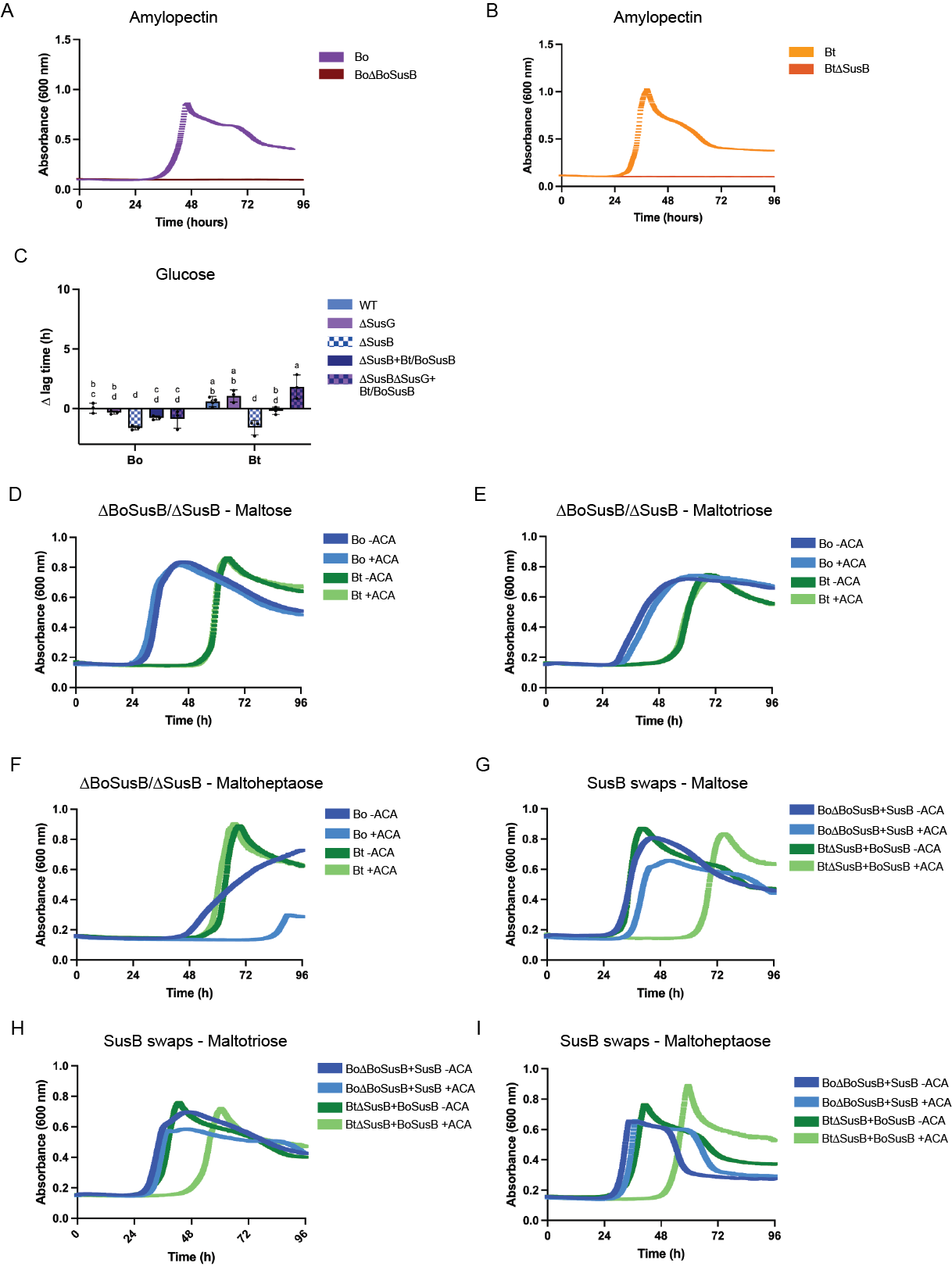


**Figure 9. Periplasmic Sus GH97 enzymes are the primary acarbose target but do not explain the different Bo and Bt phenotypes in acarbose.** A,B) Bacteria were pre-grown in minimal media (MM) with glucose and back diluted into MM + 2.5 mg/ml potato amylopectin. C) Bacteria were pre-grown in MM with glucose and back diluted into MM + 2.5 mg/ml glucose with or without 50 µM acarbose. ∆SusG corresponds to Bo∆BoSusG and Bt∆SusG. ∆SusB corresponds to Bo∆BoSusB and Bt∆SusB. ∆SusG∆SusB+Bt/BoSusB and ∆SusB+Bt/BoSusB corresponds to the SusB swapped strains. The difference in time to OD_600_ of 0.3 between the treated and untreated conditions (∆ lag time) is graphed. D-I) Bacteria were pre-grown in MM with glucose and back diluted into MM + 2.5 mg/ml of the indicated oligosaccharide with or without 50 µM acarbose (ACA). Statistical analyses were performed with a two-way ANOVA. Conditions with the same letter(s) were not significantly different from one another. A cutoff of p≤0.05 was used. All growths were performed in triplicate. The mean and standard deviation are shown in C and the average of three replicates is shown in A, B, and D-I.


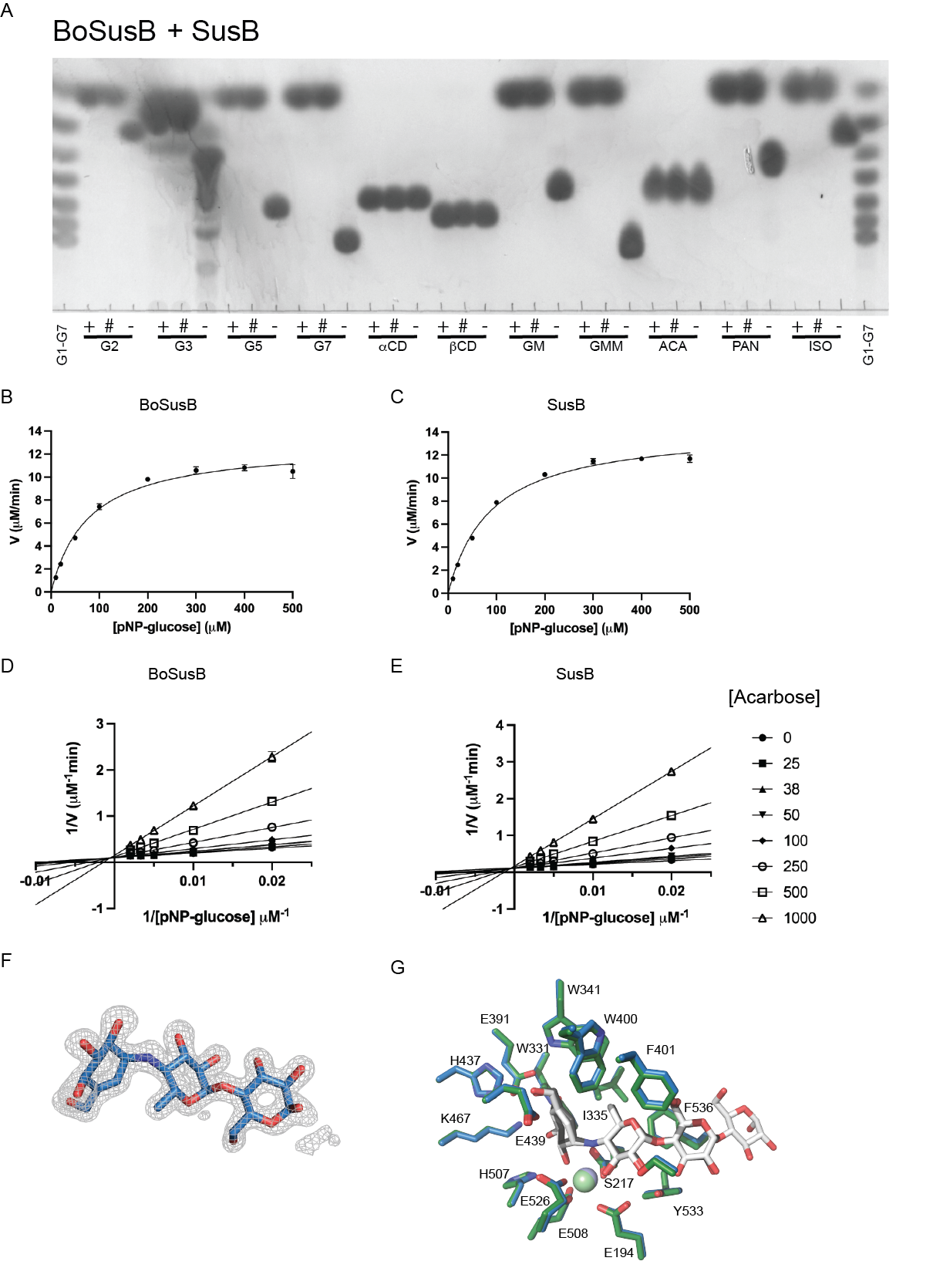


**Supplementary Figure 10. BoSusB** **and SusB are similarly inhibited by acarbose.** A) 500 nM of the indicated enzyme was incubated overnight with 5 mg/ml of the following carbohydrates: G2 – maltose; G3 – maltotriose; G5 – maltopentaose; G7 – maltoheptaose; αCD – alpha-cyclodextrin; βCD – beta-cyclodextrin; GM – 6^3^-α-d-glucosyl-maltotriose; GMM – 6^3^-α-d-glucosyl-maltotriosyl-maltotriose; ACA – acarbose; PAN – d-panose; ISO – isomaltose. + = BoGH97C_Sus_. # = SusB. - = no enzyme control.B,C) Enzymes were incubated for 10 min in various concentrations of pNP-glucose in duplicate. Initial rate as a function of substrate concentration is shown. D,E) Enzymes were pre-incubated for 10 min with acarbose before mixing with various pNP-glucose concentrations. Initial rates were recorded and used to make the double reciprocal Lineweaver-Burk plots shown. Michaelis-Menten and inhibition parameters can be found in Table 4. 10 nM of each enzyme was used for both experiments. F) F_o_-F_c_ density for acarviosin-glucose bound to chain A in the BoSusB model. While there was some density for a second glucose, an entire acarbose molecule could not be modelled in accurately. Density was contoured to 3σ. G) Comparison of acarbose bound to SusB and acarviosin-glucose bound to BoSusB. The acarviosin-glucose sticks are black and BoSusB side chains in blue. The acarbose sticks are white and SusB side chains are in green (PDB ID: 2ZQ0, (1). Bound Ca^2+^ is blue (BoSusB) and green (SusB). The superpose command in PyMOL was used to overlay the structures with a root mean squared deviation (rmsd) of 0.46 Å over all atoms. E and F were rendered in PyMOL (2).


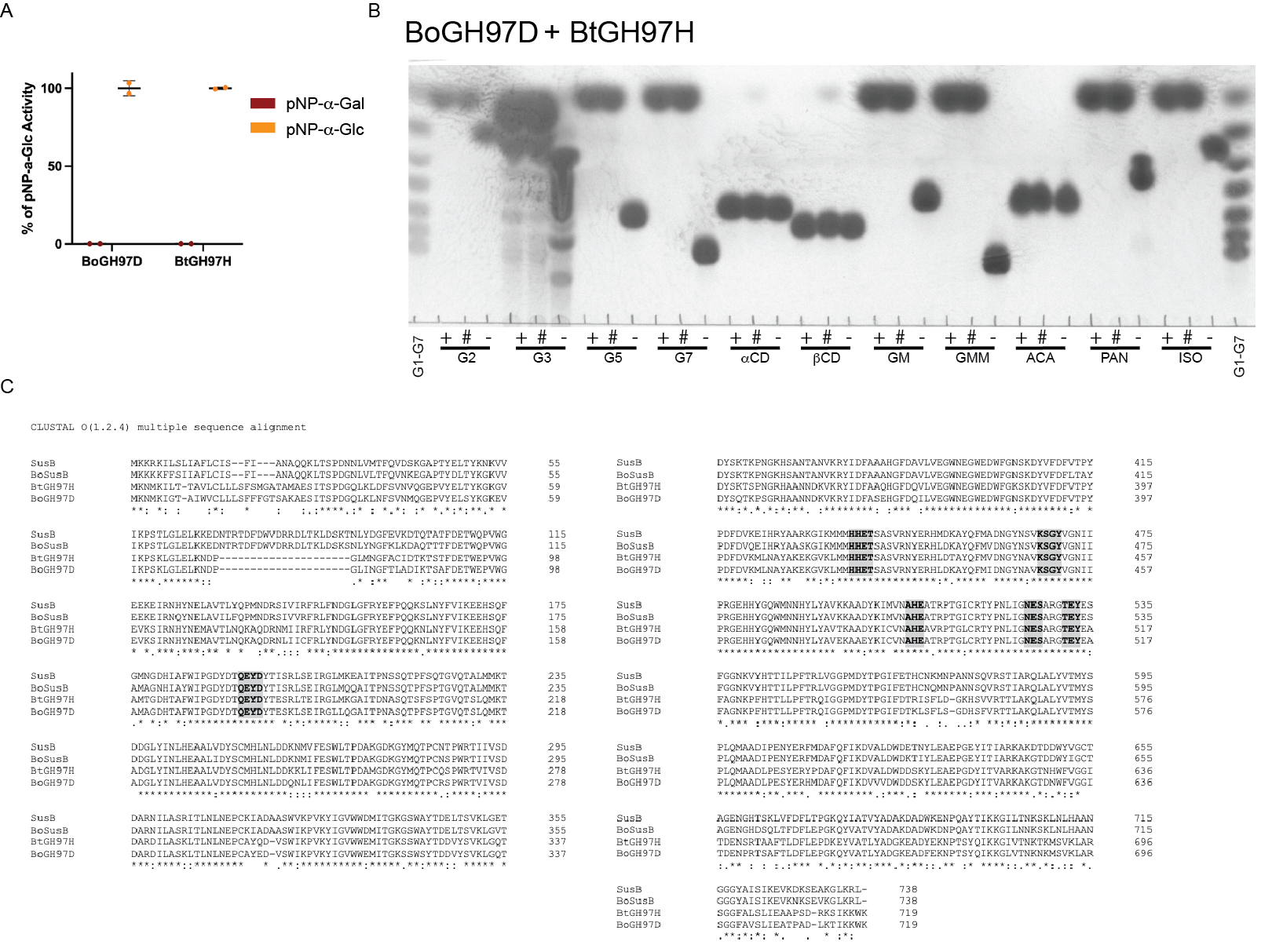


**Supplementary Figure 11. BoGH97D and BtGH97H are likely inverting α-glucosidases/glucoamylases.** A) 10 nM of each enzyme was incubated with 1 µM pNP-α-galactose (pNP-α-Gal) or pNP-α-glucose (pNP-α-Glc) and initial rates were recorded over ten minutes. Percent of pNP-α-Glc activity is graphed. B) 500 nM of the indicated enzyme was incubated overnight with 5 mg/ml of the following carbohydrates: G2 – maltose; G3 – maltotriose; G5 – maltopentaose; G7 – maltoheptaose; αCD – alpha-cyclodextrin; βCD – beta-cyclodextrin; GM – 6^3^-α-d-glucosyl-maltotriose; GMM – 6^3^-α-d-glucosyl-maltotriosyl-maltotriose; ACA – acarbose; PAN – d-panose; ISO – isomaltose. + = BoGH97C_Sus_. # = SusB. - = no enzyme control. C) The indicate enzyme amino acid sequences were aligned using ClustalOmega in the EMBL-EBI server (3). Amino acid signatures of inverting GH97s according to (4) are highlighted in grey.


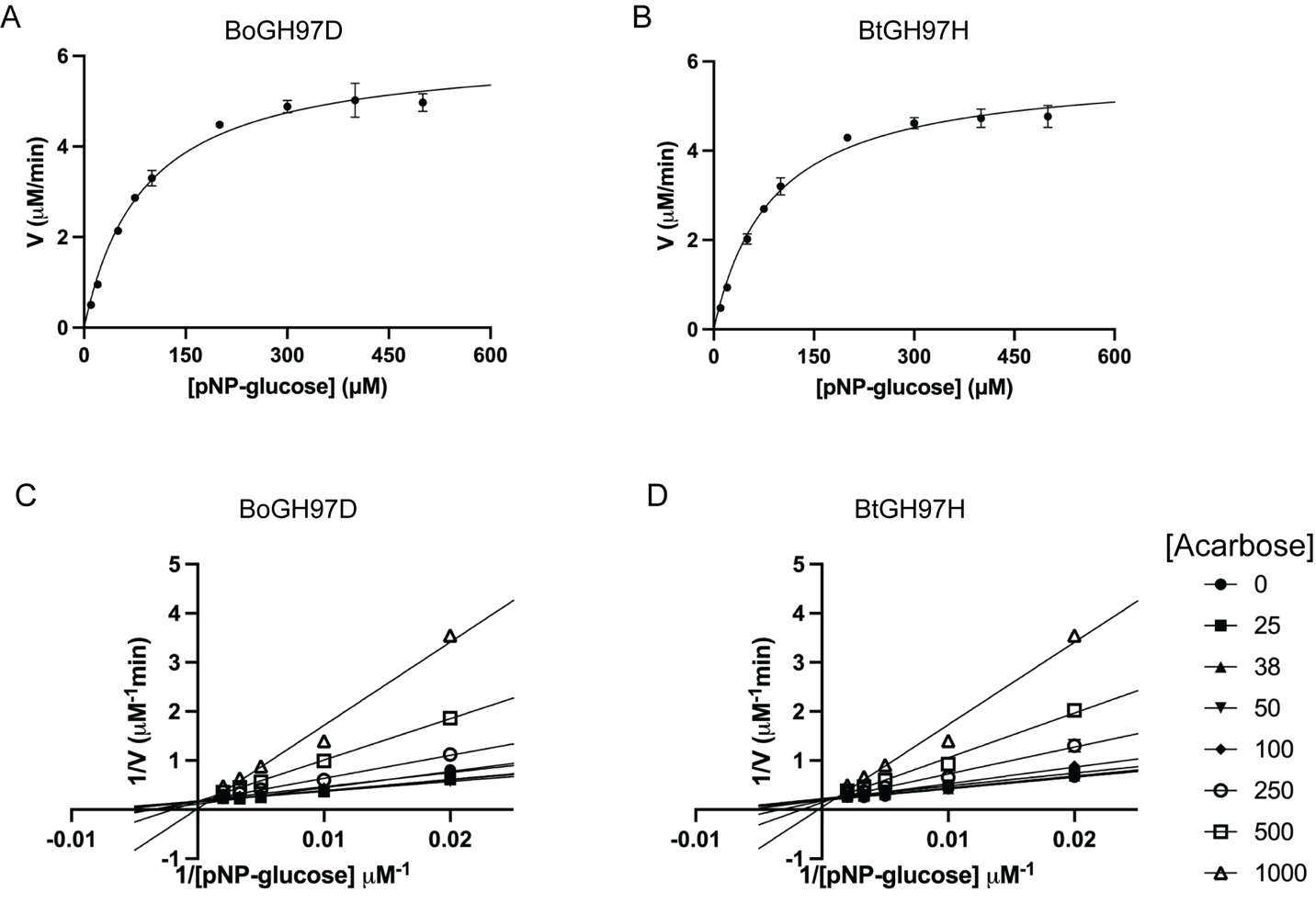


**Supplementary Figure 12. BoGH97D** **and BtGH97H are similarly inhibited by acarbose.** A,B) Enzymes were incubated for 10 min in various concentrations of pNP-glucose in duplicate. Initial rate as a function of substrate concentration is shown. C,D) Enzymes were pre-incubated for 10 min with acarbose before mixing with various pNP-glucose concentrations. Initial rates were recorded and used to make the double reciprocal Lineweaver-Burk plots shown. Michaelis-Menten and inhibition parameters can be found in Table 4. 2 nM of each enzyme was used for both experiments.


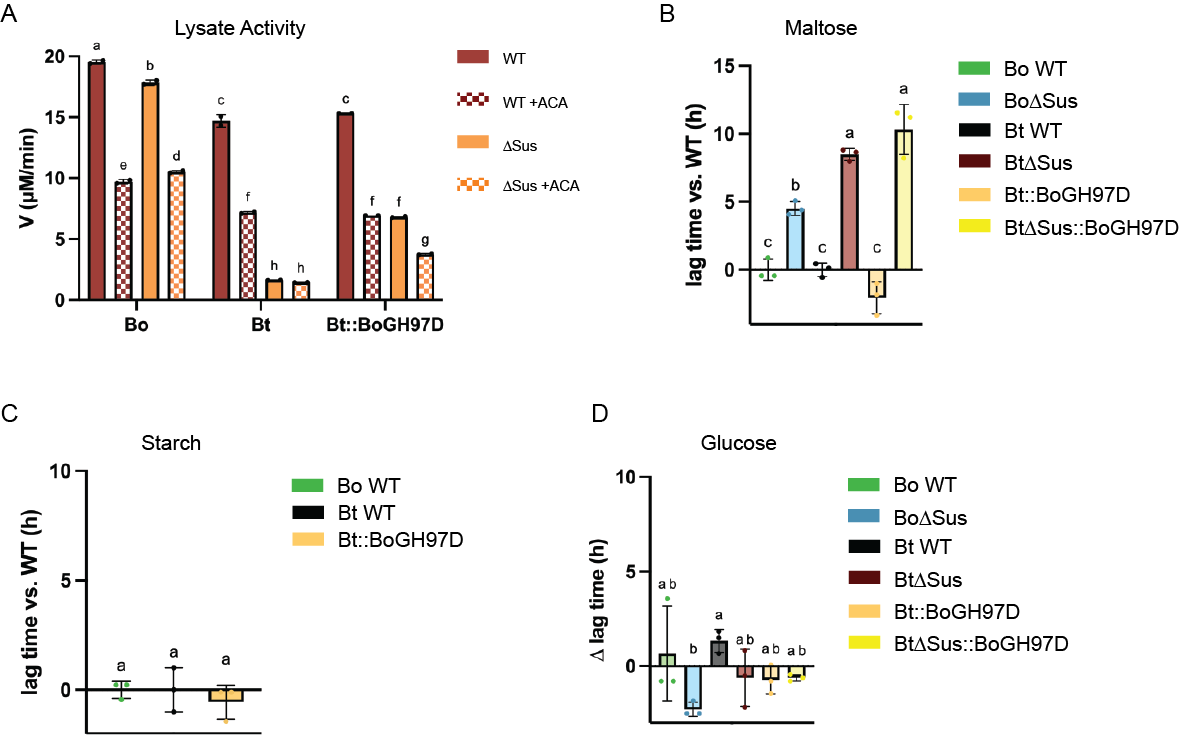


**Supplementary Figure 13. BoGH97D does not rescue the acarbose induced growth lag when expressed by Bt.** A) WT and ∆Sus strains of Bo and Bt (with or without expressing BoGH97D from a constitutive promoter) were grown in minimal media (MM) + 5 mg/ml maltose to the same OD_600_ and pelleted. Pellets were washed in PBS and cells were sonicated to release contents. Lysates were assayed in 1 mM pNP-Glc with or without 1 µM acarbose (ACA). B,C) Bacteria were pre-grown in MM with 5 mg/ml maltose and back diluted into MM + 2.5 mg/ml maltose or starch. Difference in time to OD_600_ of 0.3 between the WT and mutant strains are graphed. D) Bacteria were pre-grown as in B/C and then grown in MM + 2.5 mg/ml glucose with or without 50 µM acarbose. Acarbose induced lag times are graphed. All statistical analyses were performed with a two-way ANOVA. Conditions with the same letter(s) were not significantly different from one another. A cutoff of p≤0.05 was used.

**
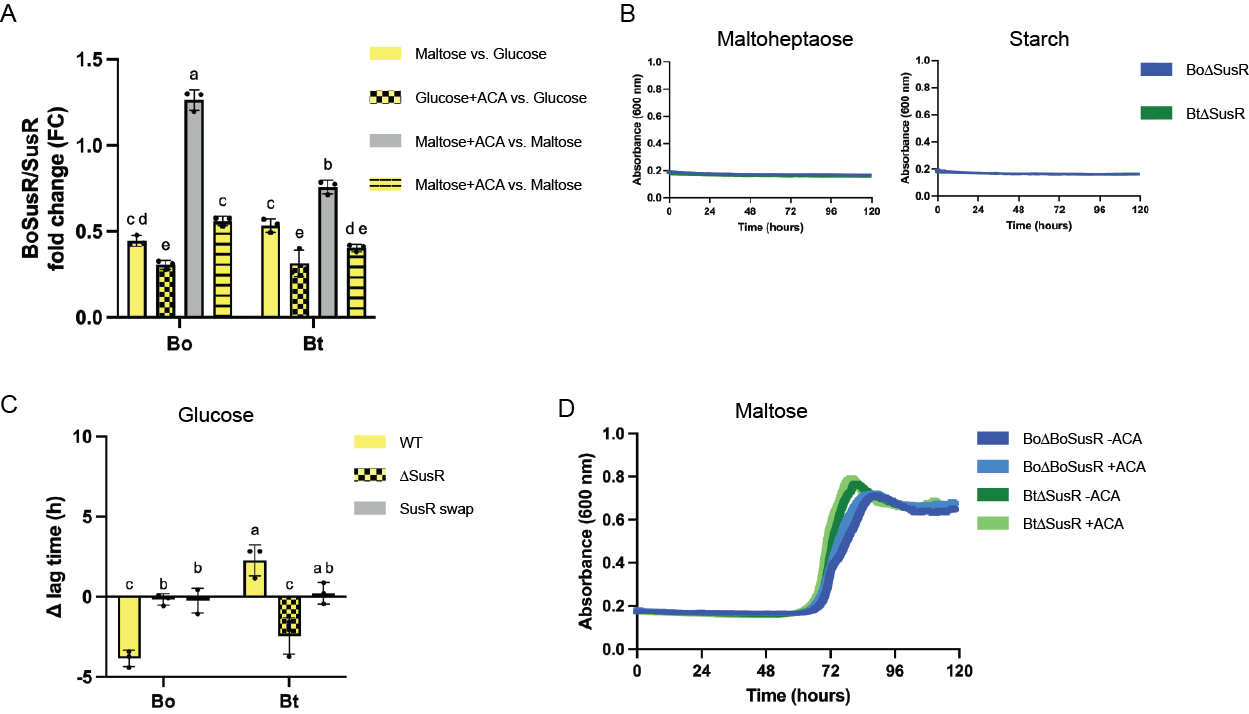
**

**Supplementary Figure 14. SusR proteins are not the source of the acarbose induced ∆ lag phenotype.**

A) Bo and Bt were grown in triplicate in minimal media (MM) with 5 mg/ml glucose or maltose with or without 50 µM acarbose and total RNA was purified. qPCR was performed to determine the fold change in BoSusR and SusR expression in the following conditions: maltose vs. glucose; glucose + 50 µM acarbose (ACA) vs. glucose; maltose + 50 µM acarbose (ACA) vs. maltose; maltose + 50 µM acarbose (ACA) vs. glucose. Statistical analyses were performed with a two-way ANOVA. Conditions with the same letter(s) were not significantly different from one another. A cutoff of p≤0.05 was used. B-D) Bo, Bt, and strains thereof were grown in MM plus glucose and back diluted into MM with 2.5 mg/ml of the indicated substrates with or without 50 µM acarbose. Acarbose induced lag times (∆ lag) are graphed. All statistical analyses were performed with a two-way ANOVA. Conditions with the same letter(s) were not significantly different from one another. A cutoff of p≤0.05 was used. Growths were performed in triplicate.

**References**

1. Kitamura M, Okuyama M, Tanzawa F, Mori H, Kitago Y, Watanabe N, Kimura A, Tanaka I, Yao M. 2008. Structural and Functional Analysis of a Glycoside Hydrolase Family 97 Enzyme from *Bacteroides thetaiotaomicron*. J Biol Chem 283:36328-36337.

2. Anonymous. The PyMOL Molecular Graphics System, v3.0.3. Schrödinger, LLC,

3. Madeira F, Pearce M, Tivey ARN, Basutkar P, Lee J, Edbali O, Madhusoodanan N, Kolesnikov A, Lopez R. 2022. Search and sequence analysis tools services from EMBL-EBI in 2022. Nucleic Acids Res 50:W276-9.

4. Gloster TM, Turkenburg JP, Potts JR, Henrissat B, Davies GJ. 2008. Divergence of catalytic mechanism within a glycosidase family provides insight into evolution of carbohydrate metabolism by human gut flora. Chem Biol 15:1058-67.
