# Supplementary material for: Acarbose impairs gut Bacteroides growth by targeting intracellular glucosidases
Source: mBio. 2024 Nov 20;15(12):e01506-24. doi: 10.1128/mbio.01506-24 (PMC11633381; doi:10.1128/mbio.01506-24)
Supplement: Legends — for supplemental tables. [file mbio.01506-24-s0002.docx]

**Table S1:** BoSusA and SusA ITC with Acarbose.

**Table S2:** BoSusB X-ray data and refinement statistics.

**Table S3:** Proteomic identification of proteins within the α-glucosidase active fraction of *B. ovatus* ΔSus lysates, determined via LC MS/MS.

**Table S4:** RNAseq of WT and ∆Sus strains of Bo grown in minimal media plus maltose (G2).

**Table S5:** RNAseq of WT and ∆Sus strains of Bt grown in minimal media plus maltose (G2).

**Table S6:** Bacterial strains used in this study.

**Table S7:** Oligonucleotide primers used in this study.

**References**

1. Kitamura M, Okuyama M, Tanzawa F, Mori H, Kitago Y, Watanabe N, Kimura A, Tanaka I, Yao M. 2008. Structural and Functional Analysis of a Glycoside Hydrolase Family 97 Enzyme from *Bacteroides thetaiotaomicron*. J Biol Chem 283:36328-36337.

2. Anonymous. The PyMOL Molecular Graphics System, v3.0.3. Schrödinger, LLC,

3. Madeira F, Pearce M, Tivey ARN, Basutkar P, Lee J, Edbali O, Madhusoodanan N, Kolesnikov A, Lopez R. 2022. Search and sequence analysis tools services from EMBL-EBI in 2022. Nucleic Acids Res 50:W276-9.

4. Gloster TM, Turkenburg JP, Potts JR, Henrissat B, Davies GJ. 2008. Divergence of catalytic mechanism within a glycosidase family provides insight into evolution of carbohydrate metabolism by human gut flora. Chem Biol 15:1058-67.
