# Supplementary material for: Acarbose impairs gut Bacteroides growth by targeting intracellular glucosidases
Source: mBio. 2024 Nov 20;15(12):e01506-24. doi: 10.1128/mbio.01506-24 (PMC11633381; doi:10.1128/mbio.01506-24)
Supplement: Table S1 — BoSusA and SusA ITC with acarbose. [file mbio.01506-24-s0003.docx]

**Supplementary Table 1.** BoSusA and SusA ITC with Acarbose

| **Protein** | ***K*_d_^1^ (μM)** | **N^1^** | ***K*_d_^2^ (μM)** | **N^2^** |
| --- | --- | --- | --- | --- |
| BoSusA | 0.94 ± 0.23 | 10.2 ± 0.1 | 0.87 ± 0.16 | 10.1 ± 0.5 |
| BoSusA D331N | 104 ± 12 | 1.5 ± 0.3 | NA | NA |
| SusA | 0.85 ± 0.23 | 9.7 ± 0.3 | 0.96 ± 0.16 | 9.9 ± 0.3 |
| SusA D331N | 72.9 ± 10 | 3.3 ± 0.6 | NA | NA |
