# Supplementary material for: Acarbose impairs gut Bacteroides growth by targeting intracellular glucosidases
Source: mBio. 2024 Nov 20;15(12):e01506-24. doi: 10.1128/mbio.01506-24 (PMC11633381; doi:10.1128/mbio.01506-24)
Supplement: Table S2 — BoSusB X-ray data and refinement statistics. [file mbio.01506-24-s0004.docx]

**Supplementary Table 2.** BoSusB X-ray data and refinement statistics.

| **Parameter** | **BoGH97C_Sus_ (BoSusB) - acarbose** |
| --- | --- |
| **PDB Code** | 9BS5 |
| **Wavelength (Å)** | 0.979 |
| **Resolution range (Å)** | 51.26 - 1.46 (1.48 - 1.46) |
| **Space group** | *P* 2_1_ 2_1_ 2_1_ |
| **Unit cell** | *a* = 107.86 Å  *b* = 116.53 Å  *c* = 144.00 Å |
| **Total reflections** | 1,799,942 (53,867) |
| **Unique reflections** | 312,566 (10,323) |
| **Multiplicity** | 5.8 (5.2) |
| **Completeness (%)** | 99.8 (99.8) |
| **Mean *I*/σ(*I*)** | 5.69 (0.20) |
| **Wilson *B*-factor (Å^2^)** | 7.7 |
| ***R*-merge** | 0.100 (2.90) |
| ***R*-meas** | 0.113 (3.2) |
| ***R*-pim** | 0.047 (1.36) |
| **CC1/2** | 0.998 (0.524) |
| **CC*** | 1 (0.829) |
| **Reflections used in refinement** | 311,960 (10,320) |
| **Reflections used for *R*-free** | 158,61 (530) |
| ***R*-work** | 0.204 (0.447) |
| ***R*-free** | 0.224 (0.451) |
| **Number of non-hydrogen atoms** | 12,684 |
| **macromolecules** | 11,035 |
| **ligands** | 157 |
| **solvent** | 1,492 |
| **Protein residues** | 1,377 |
| **RMS (bonds, Å)** | 0.013 |
| **RMS(angles, °)** | 1.52 |
| **Ramachandran favored (%)** | 97.0 |
| **Ramachandran allowed (%)** | 3.0 |
| **Ramachandran outliers (%)** | 0 |
| **Rotamer outliers (%)** | 0.5 |
| **Clashscore** | 0.8 |
| **Average B-factor (Å^2^)** | 17.0 |
| **Macromolecules (Å^2^)** | 15.0 |
| **Ligands (Å^2^)** | 22.4 |
| **Solvent (Å^2^)** | 31.3 |

Data for highest resolution bin are in parentheses.
