# Supplementary material for: Acarbose impairs gut Bacteroides growth by targeting intracellular glucosidases
Source: mBio. 2024 Nov 20;15(12):e01506-24. doi: 10.1128/mbio.01506-24 (PMC11633381; doi:10.1128/mbio.01506-24)
Supplement: Supplemental tables — Tables S6 and S7. [file mbio.01506-24-s0008.docx]

**Supplementary Table 6.** Bacterial strains used in this study.

| **Strain** | **Genotype** | **Reference** |
| --- | --- | --- |
| Bo | Bo∆*tdk* (BoΔ*bovatus_02336*) | (72) |
| Bt | Bt∆*tdk* (BtΔ*bt2275*) | (30) |
| Bo∆BoSusG | Bo∆*tdk*∆*bovatus_03803* | (47) |
| Bt∆SusG | Bt∆*tdk*∆*bt3698* | (31) |
| Bo∆BoSusC | Bo∆*tdk*∆*bovatus_3807* | This study |
| Bt∆SusC | Bt∆*tdk*∆*bt3702* | (31) |
| Bo∆BoSusC∆BoSusG | Bo∆*tdk*∆*bovatus_03803,* ∆*bovatus_03807* | This study |
| Bt∆SusCG | Bt∆*tdk*∆*bt3698*∆*bt3702* | (32) |
| BoΔBoBoSusG+SusG | BoΔ*tdk*Δ*bovatus_03803*+*bt3698* | (47) |
| BtΔSusG+BoSusG | BtΔ*tdk*Δ*bt3698*+*bovatus_03803* | (47) |
| Bo∆BoSusA | Bo∆*tdk*, *bovatus_03809leu5🡪stop* | This study |
| Bt∆SusA | Bt∆*tdk, bt3704leu5🡪stop* | This study |
| Bo∆BoSusB | Bo∆*tdk*, *bovatus_03808ser8🡪stop* | This study |
| Bt∆SusB | Bt∆*tdk, bt3703ser8🡪stop* | This study |
| Bo∆BoSusB+SusB | Bo∆*tdk*∆*bovatus_03808+bt3703* | This study |
| Bo∆BoSusB∆BoSusG+SusB | Bo∆*tdk*∆*bovatus_03803*∆*bovatus_03808*+*bt3703* | This study |
| Bt∆SusB+BoSusB | Bt∆*tdk∆bt3703*+*bovatus_03808* | This study |
| Bt∆SusB∆SusG+BoSusB | Bt∆*tdk*∆*bt3698∆bt3703*+*bovatus_03808* | This study |
| Bo∆Sus | Bo∆*tdk*∆*bovatus_03803-bovatus03809* | N/A |
| Bt∆Sus | Bt∆*tdk∆bt3698-bt3704* | N/A |
| Bt::BoGH97D | Bt∆*tdk*::*bovatus_04772* | This study |
| Bt∆Sus::BoGH97D | Bt∆*tdk*∆*bt3698-bt3704::bovatus_04772* | This study |
| Bo∆BoSusR | Bo∆*tdk*∆*bovatus_03810* | This study |
| Bo∆BoSusR+BtSusR | Bo∆*tdk*∆*bovatus_03810*+*bt3705* | This study |
| Bt∆SusR | Bt∆*tdk*∆*bt3705* | This study |
| Bt∆SusR+BoSusR | Bt∆*tdk*∆*bt3705*+*bovatus_03810* | This study |

**Supplementary Table 7.** Oligonucleotide primers used in this study.

| **Primer Name** | **Sequence (5’-3’)** | **Function** |
| --- | --- | --- |
| SusAstop FlankF | GCGGTCGACGGAGGGCATATCTTTAAACAGATAG | SusA stop (∆SusA) |
| SusAstop FlankR | GTTACCATGAAAAGGAAT**TAA**TTATTCATTATC | SusA stop (∆SusA) |
| SusR FlankF | GATAATGAATAA**TTA**ATTCCTTTTCATGGTAACTAGATTTTTAAAATTTGTTCGGGGG | SusA stop (∆SusA) |
| SusR FlankR | GCGTCTAGACGGTTTTGTTTGTCGTATAATCAGCG | SusA stop (∆SusA) |
| SusBstop FlankF | GCGGTCGACGCATACATGAGTAATCAACCAAAGC | SusB stop (∆SusB) |
| SusBstop FlankR | GAAAAAGAGAAAGATTTTA**TAG**CTCATCGC | SusB stop (∆SusB) |
| SusA FlankF | GCGATGAG**CTA**TAAAATCTTTCTCTTTTTCATTCTATTTATGGTATTAAATTATAAGC | SusB stop (∆SusB) |
| SusA FlankR | GCGGCCGCCCTTCCTCGACAATCACGATACTTC | SusB stop (∆SusB) |
| BoGH13B_Sus_stop FlankF | GCGGTCGACGGCAGGCATGTCTTTGAATAGATAATTG | BoSusA stop (∆BoSusA) |
| BoGH13B_Sus_stop FlankR | CCATGAAACGAAAT**TAG**TTATTTGC | BoSusA stop  (∆BoSusA) |
| BoSusR FlankF | GCAAATAA**CTA**ATTTCGTTTCATGGTAGCTAGATTTTTAACTGTTTGCTCGG | BoSusA stop (∆BoSusA) |
| BoSusR FlankR | GCGTCTAGAGGTCGTATAATCTGCTAACCACCC | BoSusA stop (∆BoSusA) |
| BoGH97C_Sus_stop FlankF | GCGGTCGACGAGGTGCATACAAGAATAATC | BoSusB stop  (∆BoSusB) |
| BoGH97C_Sus_stop FlankR | GAATGAAAAAGAAAAAGTTTTTT**TAG**ATCATCGC | BoSusB stop (∆BoSusB) |
| BoGH13B_Sus_ FlankF | GCGATGAT**CTA**AAAAAACTTTTTCTTTTTCATTCTATTTATGGTATTAAATTGTAAGC | BoSusB stop (∆BoSusB) |
| BoGH13B_Sus_ FlankR | GCGTCTAGAGCTGACCTTCCTCGATAATCACG | BoSusB stop (∆BoSusB) |
| BoGH97C_Sus_ FlankF | GCGGCCGCCCCAATCTGATCGGTAATGAATCCGC | ∆BoSusC |
| BoGH97C_Sus_ FlankR | GATAACAAATTTAACTTGCTTCATGC | ∆BoSusC |
| BoSusD FlankF | GCATGAAGCAAGTTAAATTTGTTATCAGTTAAGATTGTAATTTATTAAAAAGTAGAC | ∆BoSusC |
| BoSusD FlankR | GCGTCTAGACGCGGTTGTCCGGTGTATACTTCGGC | ∆BoSusC |
| SusAflFwd | GCGGTCGACGACCTTCCTCGACAATCACGATAC | Full ∆SusB/GH97swap |
| SusAflRev | CGATAAAATCTTTCTCTTTTTCATTC | Full ∆SusB |
| SusCflFwd | GAATGAAAAAGAGAAAGATTTTATCGTAATAAGGAAAAGAAATAACAAAGATAGAG | Full ∆SusB |
| SusCflRev | GCGGCCGCCCTTCATTGTCAATAGCCAGACCG | Full ∆SusB/GH97swap |
| SusAflRev4swap | CTATTTATGGTATTAAATTATAAGCTAAC | GH97 swap – BoSusB into Bt |
| SusABoGH97C_Sus_ overlap | GTTAGCTTATAATTTAATACCATAAATAGAATGAAAAAGAAAAAGTTTTTTTCGATCATC | GH97 swap – BoSusB into Bt |
| BoGH97C_Sus_ Rev pEx | CCTTATTATAGTCTTTTCAATCCC | GH97 swap – BoSusB into Bt |
| BoGH97C_Sus_SusC overlap | GGGATTGAAAAGACTATAATAAGGAAAAGAAATAACAAAGATAGAGAATATACATTTTGG | GH97 swap – BoSusB into Bt |
| BoGH13B_Sus_flFwd | GCGGTCGACGTTTCTACCGTTCGGAGGAAGATAC | Full ∆BoSusB/ GH97 swap |
| BoGH13B_Sus_flRev | CGAAAAAAACTTTTTCTTTTTCATTCTATTTATGG | Full ∆BoSusB |
| BoSusCflFwd | CATAAATAGAATGAAAAAGAAAAAGTTTTTTTCGTAAATAAAGGATAGTCTTAAAAAAAG | Full ∆BoSusB |
| BoSusCflRev | GCGTCTAGACCATTGCCAAAGGATTAGTCAAAC | Full ∆BoSusB/ GH97 swap |
| BoGH13B_Sus_ flrev4swap | CTATTTATGGTATTAAATTGTAAGCTAACCG | GH97 swap – SusB into Bo |
| BoGH13B_Sus_SusB overlap | CGGTTAGCTTACAATTTAATACCATAAATAGAATGAAAAAGAGAAAGATTTTATCGCTC | GH97 swap – SusB into Bo |
| SusBrevpEx | CCTTTATTTATAATCTTTTCAAACCTTTAGC | GH97 swap – SusB into Bo |
| SusBBoSusC overlap | GCTAAAGGTTTGAAAAGATTATAAATAAAGGATAGTCTTAAAAAAAGTGTTTTCAATAGG | GH97 swap – SusB into Bo |
| SusApETFwd | CATCATCACCACCATCACGAGAACCTGTACTTCCAGGGCGCAACAAGCACTATAAAG | N-terminal 6x His-Tev construct in pETite |
| SusApETRev | GTGGCGGCCGCTCTATTATTAAAAGGATAAAAGGTATATTTCACG | N-terminal 6x His-Tev construct in pETite |
| SusBpETFwd | CATCATCACCACCATCACCAACAGAAATTAACCTCACCGGAC | N-terminal 6x His construct in pETite |
| SusBpETRev | GTGGCGGCCGCTCTATTATTATAATCTTTTCAAACCTTTAGCTTC | N-terminal 6x His construct in pETite |
| BoGH13B_Sus_pET Fwd | CATCATCACCACCATCACGAGAACCTGTACTTCCAGGGCGCAACCAATATAAAGAAAGTG | N-terminal 6x His-Tev construct in pETite |
| BoGH13B_Sus_pET Rev | GTGGCGGCCGCTCTATTATTAGAAGGCTAAAAGGTATATCCC | N-terminal 6x His-Tev construct in pETite |
| BoGH97C_Sus_pET Fwd | CATCATCACCACCATCACCAACAGAAACTGACCTCACCGGATG | N-terminal 6x His construct in pETite |
| BoGH97C_Sus_pET Rev | GTGGCGGCCGCTCTATTATTATAGTCTTTTCAATCCCTTAACC | N-terminal 6x His construct in pETite |
| SusA D331N Fwd | CATCCGTCAG**AAC**ACACACCCTTATG | D331N mutation |
| SusA D331N Rev | CATAAGGGTGTGT**GTT**CTGACGGATG | D331N mutation |
| BoGH13B_Sus_ D331 Fwd | GAATCCGCCAG**AAC**ACACATCCTTAC | D331N mutation |
| BoGH13B_Sus_ D331 Rev | GTAAGGATGTGT**GTT**CTGGCGGATTC | D331N mutation |
| BtGH97H/BoGH97D Fwd | CATCATCACCACCATCACGAAAGTATCACTTCTCCTGAC | N-terminal 6x His construct in pETite |
| BtGH97H Rev | GTGGCGGCCGCTCTATTATTTCCATTTCTTAATTGATTTCCGG | N-terminal 6x His construct in pETite |
| BoGH97D Fwd | GTGGCGGCCGCTCTATTATTTCCATTTCTTGATCGTTTTCAAG | N-terminal 6x His construct in pETite |
| Bovatus_04772 pNBU2 Fwd | GCGCATATGATGAAGAACATGAAAATAGGAACTG | bovatus_04772 pNBU2 plasmid |
| Bovatus_04772 pNBU2 Rev | GCGTCTAGATTATTTCCATTTCTTGATCGTTTTCAAG | bovatus_04772 pNBU2 plasmid |
| Bt/Bo 16S qPCR | GGTAGTCCACACAGTAAACGATGAA | 16S rRNA normalization |
| Bt/Bo 16S qPCR | CCCGTCAATTCCTTTGAGTTTC | 16S rRNA normalization |
| Bovatus_04772 qPCR | CGGCGATCATACGGCATTCTG | Bovatus_04772 (BoGH97D) transcript |
| Bovatus_04772 qPCR | ACCCGTCGGCTGTCTTCATTTG | Bovatus_04772 (BoGH97D) transcript |
| Ovatus F susC qPCR | AACGCAGGTTTCGACTTTGC | Bovatus_03807 (BoSusC) transcript |
| Ovatus R susC qPCR | ATGGTGAATTCAAGCCCCGT | Bovatus_03807 (BoSusC) transcript |
| Bt4581 F qPCR | CCGGTGTGGGGAGAAGTGAAAT | Bt4581 (BtGH97H) transcript |
| Bt4581 R qPCR | TCCCGGAATCCAAAAGGCAGTAT | Bt4581 (BtGH97H) transcript |
| Bt susC qPCR | GCTATTGGCGGGGCATTGG | Bt3702 (SusC) transcript |
| Bt susC qPCR | CAGCGGATTTTGGGGAGAGTTCG | Bt3702 (SusC) transcript |
| dBoSusR-bo03811flankFSalI | GGCGTCGACGCCTTATAGTCATTGTAACCACGTGC | ∆BoSusR |
| dBoSusR-bo03811flankR | GCAAATAACAAATTTCGTTTCATTGTTATTCACAGATAGCAATATGTTATTACTTC | ∆BoSusR |
| dBoSusR-BoSusAflankF | CAATGAAACGAAATTTGTTATTTGCTATTTTAC | ∆BoSusR |
| dBoSusR-BoSusAflankRXbaI | GGCTCTAGAGAATAGATAATTGTTCGAACCGCAATG | ∆BoSusR |
| BtSusRinBo-BtSusRFSalI | GGCGTCGACTTACAAATTCATTACATCCTGTTCGAACCG | SusR swap – SusR into Bo |
| BtSusRinBo-BtSusRR | GGTAACTAGATTTTTAAAATTTGTTCGGGGG | SusR swap – SusR into Bo |
| BtSusRinBo-BoSusAflankF | GAACAAATTTTAAAAATCTAGTTACCATGAAACGAAATTTGTTATTTGCTATTTTAC | SusR swap – SusR into Bo |
| BtSusRseqF | GTCATCCAGTTTTTTAAGGGCCG | Screen for SusR into Bo |
| BoSusAseqR | CAAAGTCGGCGTAAGGATGTGTATC | Screen for SusR into Bo |
| dSusR-bt3706FlankFSalI | GGCGTCGACGCTCAAATAAATATGTAGAGAGGC | ∆SusR |
| dSusR-bt3706flankR | GAATAATAAATTCCTTTTCATGTACACTCTAGTCTCTCTTCATGTCTCCAATAGAC | ∆SusR |
| dSusR-SusAflankF | GTACATGAAAAGGAATTTATTATTCATTATC | ∆SusR |
| dSusR-SusAflankRXbaI | GGCTCTAGAGGGCATATCTTTAAACAGATAGTTGTCC | ∆SusR |
| BoSusRinBt-SusRFSalI | GGCGTCGACTTATAGGTTCATTACATTCTGCTCGAAC | SusR swap – BoSusR into Bt |
| BoSusRinBt-BoSusRF | GGTAGCTAGATTTTTAACTGTTTGC | SusR swap – BoSusR into Bt |
| BoSusRinBt-SusAflankF | GCAAACAGTTAAAAATCTAGCTACCATGAAAAGGAATTTATTATTCATTATCTTAC | SusR swap – BoSusR into Bt |
| BoSusRseqF | GTAGAATGGGCAAGTTCTAATTTCAAG | Screen for BoSusR into Bt |
| BtSusAseqR | CAATCCACCAGATACTGCTC | Screen for BoSusR into Bt |
| bovatus_03810 bosusR qPCR FWD | GGCAGACTCGTCCTTATATT | Bovatus_03810 (BoSusR) transcript |
| bovatus_03810 bosusR qPCR REV | TTCCCATTACTCCCATTACC | Bovatus_03810 (BoSusR) transcript |
| Bt3705 susR qPCR FWD | CTGTTTCTCCTCATTGCTATTT | Bt3705 (SusR) transcript |
| Bt3705 susR qPCR REV | CCGGTCTGTTCCAATTCT | Bt3705 (SusR) transcript |

Restriction sites are highlighted in red. Mutagenic nucleotides are **bolded**.
